# Management of systemic lupus erythematosus with kidney involvement: systematic literature review to inform the 2025 update of EULAR recommendations

**DOI:** 10.1016/j.ero.2025.07.006

**Published:** 2025-08-23

**Authors:** Myrto Kostopoulou, George Bertsias, Carlo Alberto Scire, Dimitrios T. Boumpas, Antonis Fanouriakis

**Affiliations:** 1 Department of Nephrology, “G. Gennimatas” General Hospital, Athens, Greece; 2 Rheumatology, Clinical Immunology and Allergy, University Hospital of Heraklion, Greece; 3 Epidemiology Research Unit, Italian Society of Rheumatology, Milan, and School of Medicine and Surgery, University of Milan-Bicocca, Milan, Italy; 4 Rheumatology and Clinical Immunology Unit, “Attikon” University Hospital, National Kapodistrian University of Athens, Greece; 5 Laboratory of Autoimmunity and Inflammation, Biomedical Research Foundation of the Academy of Athens, Athens, Greece; 6 Joint Academic Rheumatology Program, Medical School, National and Kapodistrian University of Athens, Athens, Greece

## Abstract

**Objectives:**

To identify, appraise, and synthesise current evidence regarding the management of systemic lupus erythematosus (SLE) with kidney involvement towards informing the 2025 update of the European Alliance of Associations for Rheumatology (EULAR) recommendations.

**Methods:**

The Task Force formulated 11 research questions grouped into 6 distinct sections, namely indications of diagnostic kidney biopsy; treatment targets; efficacy and safety of immune treatments in lupus nephritis (LN); efficacy and safety of nonimmune treatments; treatment duration; and indications for repeat kidney biopsy. A dedicated systematic literature review (SLR) was performed for each section. SLRs were performed in PubMed, Embase, and the Cochrane Central Register of Controlled Trials from January 2019 to March 2024.

**Results:**

Patients with SLE with low-grade proteinuria (<500-1000 mg/d) and/or haematuria may have histologically active disease, based on evidence of moderate quality. Observational studies of good quality have demonstrated an association between complete or partial renal response within the first 6 to 12 months and long-term kidney survival. A combination of 2 immunosuppressive agents, such as belimumab with mycophenolate mofetil (MMF)/cyclophosphamide, voclosporin with MMF, or obinutuzumab with MMF, is associated with higher response rates, based on high-quality randomised controlled trials (RCTs). Discontinuation of treatment has been linked with an increased risk of relapse, as evidenced by 3 RCTs with substantial heterogeneity.

**Conclusions:**

High-quality RCTs indicate a benefit from combination treatments compared with previous standard-of-care in LN. Patients who respond within the first year of treatment tend to have better long-term outcomes. Kidney biopsy is essential for diagnosis, but the role of repeat biopsy awaits confirmation from higher-quality studies.

## INTRODUCTION

Kidney involvement represents one of the most severe complications of systemic lupus erythematosus (SLE), with significant impact on morbidity and mortality. In 2019, the European Alliance of Associations for Rheumatology (EULAR), together with the European Renal Association-European Dialysis and Transplant Association, updated the 2012 recommendations for the management of lupus nephritis (LN) [1]. Since then, several landmark randomised controlled trials (RCTs) have been published, initiating a paradigm shift in LN management. In light of these advances, an update of the 2019 recommendations was considered timely and necessary.

The aim of this systematic literature review (SLR) was to identify, appraise, and provide the evidence for the research questions posed by a dedicated EULAR Task Force, ie, the role of diagnostic and repeat kidney biopsy, the efficacy and safety of immune and nonimmune treatments, the treatment target, and the optimal treatment duration in patients with LN. The results of this SLR were used to inform the 2025 update of the EULAR recommendations for the management of SLE with kidney involvement (submitted in parallel) [1].

## METHODS

Being an update, the current SLR retained the structure and research topics of the 2019 SLR [2]. Initially, a draft of an SLR protocol—including a list of candidate treatment-related research questions and their respective Population Intervention Comparison Outcome (PICO)—was developed by the convenor (DTB), the methodologist (GB), and the 2 fellows responsible for the SLR (AF and MK) and was sent to Task Force members for their comments and edits. Although the focus of the update was on management (both immune and nonimmune), Task Force members were encouraged to suggest additional topics which they considered important.

The final set approved by the Task Force included 11 research questions grouped into 6 distinct sections with their respective PICOs (Supplementary File S1). The sections included the following: (1) indications of diagnostic kidney biopsy; (2) efficacy and safety of immune treatments in LN (proliferative, nonproliferative LN, and thrombotic microangiopathy [TMA]); (3) efficacy and safety of nonimmune treatments (including angiotensin-converting enzyme inhibitors [ACEis], angiotensin II receptor blockers [ARBs], sodium-glucose cotransporter-2 inhibitors [SGLT2is], statins, osteoporosis-preventing treatments, and kidney replacement treatments); (4) treatment targets; (5) treatment duration; and (6) indications for repeat kidney biopsy. Additionally, the SLR of section 4 identified potential patient subgroups and prognostic factors at the time of biopsy that would allow for a risk stratification model to inform tailored treatment decisions.

Based on the above, 3 broad search strings were developed based on PICOs, which addressed all research questions (Supplementary File S1). Two fellows (AF and MK) performed the 3 SLRs independently in 3 different databases (MEDLINΕ, EMBASE, and the Cochrane Central Register of Controlled Trials). Studies in the English language, including adult or paediatric LN population (biopsy proven or not), were considered eligible. Since this was an update, studies had to be published between the completion of the previous SLR (January 2019) and May 2024. An exception was made for a single phase 3 RCT (REGENCY trial of obinutuzumab [OBI]) that was published after the aforementioned end date [3]; specifically, the Task Force decided to include this study due to the importance of its findings and potential clinical implications [3]. For research questions relating to treatment of LN (sections 2, 3, and 5), only RCTs and quasi-RCTs were considered eligible. Contrary to prognostic/screening questions (sections 1, 4, and 6), a more inclusive strategy was selected because respective RCTs are lacking, and evidence originates mainly from observational cohort studies. Case-control and cross-sectional studies, reviews, case series, case reports, conference abstracts, animal studies, trials in non-English language, and cohort studies with a sample size <100 were excluded (<20 for sections 1 and 6, as trials with kidney biopsies are generally performed in smaller numbers of patients). Eligible studies were also reviewed for additional records within their reference lists.

PICOs were used to develop dedicated forms to extract all relevant data from eligible studies. Disagreements between reviewers were resolved through discussion. Results are presented separately for each section; moreover, they are tabulated and synthesised within each section based on the intervention/exposure, the outcome of interest, and risk/prognostic factors.

Risk of bias (RoB) and study quality of each eligible study were assessed with the revised Cochrane Risk of Bias Assessment Tool for RCTs (ROB V.2) and the Newcastle-Ottawa scale for observational studies [4,5].

## RESULTS

### Section 1: Indications for diagnostic kidney biopsy

We retrieved 82 studies and screened 63 after removing duplicates (Supplementary Fig S1); 15 observational studies were considered eligible, 8 related to section 1 (indications of diagnostic biopsy), and the remaining to section 6 (role of repeat kidney biopsy).

Study characteristics for section 1 are presented in Supplementary Table S1. Seven studies explored the incidence of biopsy-proven LN in patients with SLE with low-level proteinuria (ranging from 0.2-1 g/24 hours), with or without glomerular haematuria and consistently found significant rates of proliferative (class III/IV) histologic classes. In a retrospective study (fair quality) of 84 patients with proteinuria <0.5 g/24 hours, 71.4% had glomerular lesions in kidney biopsy and 26.2% were classified as class III [6]. In 2 studies (good quality) of patients with proteinuria <1 g/24 hours, a significant proportion (38.6%-75%) had class III/IV or mixed class III/IV and class V in their kidney biopsies [7,8]. In the largest study (low quality) (242 kidney biopsies classified as proliferative or mixed LN), 35.9% had proteinuria <500 mg/24 hours at the time of biopsy (Supplementary Table S1) [9]. Nevertheless, in the single study that explored the role of proteinuria and various components of urine sediment in predicting proliferative classes, a protein level >1 g/24 hours had the best performance (area under the curve 0.806, sensitivity 82.61%, specificity 64.71%) (low quality) [10].

Three studies of low quality explored the safety of the biopsy procedure and reported low rates of severe complications (defined as transfusion or hospitalisation related to biopsy) ranging from 0.8% to 4.6%. No death was reported [[11], [12], [13]].

### Section 2: Immune treatment of LN

Section 2 included 3 subquestions related to the efficacy and safety of different treatments in various forms of LN (class III/IV, class V, and TMA). The SLR identified 1205 studies; 639 were screened for eligibility (flowchart in Supplementary Fig S2). Finally, 74 studies were identified, of which 17 RCTs, 3 extension studies, and 1 post hoc analysis were eligible for section 2 and thus were included in the qualitative synthesis. The remaining studies were eligible for sections other than section 2 (tabulated study characteristics in Supplementary Tables S2-S5).

## EFFICACY

### Calcineurin inhibitors (tacrolimus, voclosporin)

Three RCTs, all performed in Asian populations, compared the efficacy of tacrolimus (TAC)-based regimens (TAC administered as a single or as an add-on immunosuppressive [IS] treatment) with either mycophenolate mofetil (MMF), cyclophosphamide (CYC), or azathioprine (AZA) (Supplementary Table S3) [[14], [15], [16]]. Although overall response rates (complete and partial) at 24 weeks in the TAC-based arms were at least as high as the comparator arms (range 83%-90.6%), a direct comparison of the numerical response rates between studies was not appropriate due to marked variability in the treatment regimens. Specifically, TAC was compared with intravenous (iv) CYC (in addition to 0.8 mg/kg/d of prednisone) in one head-to-head RCT (299 patients, low RoB) [16], TAC combined with AZA was compared with iv CYC (plus prednisone at 0.5 mg/kg/d) in another RCT (100 patients, RoB with some concerns) [14], and TAC combined with MMF was compared with CYC combined with MMF (plus prednisone at 45 mg/d) in a third RCT (234 patients, high RoB) [15].

Regarding voclosporin (VCS), 3 trials (1 phase 2 RCT with RoB of some concerns [AURA-LV], 1 phase 3 of low RoB [AURORA 1], and an extension study of the latter [AURORA 2]), explored its short- and long-term efficacy and safety compared with placebo, in patients receiving 2 g of MMF. AURA-LV and AURORA 1 included 265 and 357 patients, respectively, with biopsy-proven class III/IV, class V, or mixed LN, a urine protein to creatinine ratio (UPCR) >1.5 mg/mg, and estimated glomerular filtration rate (eGFR) >45 mL/min/1.73 m^2^ [17,18]. Population and treatment protocols were similar between trials, except for an additional arm with a higher dose of VCS in the phase 2 trial (Table 1, Supplementary Table S2). Patients followed a strict low-dose prednisone protocol starting at 20 mg/d, tapered to 2.5 mg/d by week 16. The primary endpoint, complete renal response (defined as UPCR ≤0.5 mg/mg, eGFR ≥60 mL/min or no decrease >20% from baseline, no rescue treatment, and no >10 mg of prednisone per day for ≥3 days or for ≥7 days in total during weeks 44-52) was assessed at 24 weeks in the phase 2 trial and at 56 weeks in the phase 3 RCT. VCS had significantly better efficacy in both trials (odds ratio [OR] for standard-dose VCS 2.03; 95% CI 1.01-4.05 and OR 2.65; 95% CI 1.64-4.27 in phase 2 and phase 3, respectively). The benefit from VCS was confirmed in an integrated analysis that included pooled data from both RCTs (OR 2.76; 95% CI 1.88-4.05 at 48 weeks). AURORA 2 was a safety extension study that enrolled patients from AURORA 1 who continued their double-blind treatments for another 104 weeks following the end of AURORA 1. Although complete response (CR) was a secondary endpoint, the higher response rates in the VCS arm were maintained throughout the follow-up period (VCS: 50.9%, placebo: 39.0%; OR 1.74, 95% CI 1.00-3.03). Notably, after 3 years of continuous treatment, 23.8% in the VCS group vs 26.0 % in the placebo had a renal flare; however, there was no significant difference between arms (hazard ratio [HR] 0.85; 95% CI 0.42-1.73) [19].

**Table 1:** Comparative description of the largest RCTs in lupus nephritis.

	REGENCY	BLISS-LN	AURORA
Sample size	271	448	357
Experimental drug	Obinutuzumab	Belimumab	Voclosporin
Class V	0	16%	14%
UPCr at entry (g/g)	3.14	3.4	3.8-4.1
eGFR at entry (mL/min/1.73 m^2^)	101.9	100.5	90.4-92.1
Dosage	2 g (1 g at d1 d15) Repeat 2 g (6m) Group 1: Repeat 1 g at 12 m Group 2: Repeat 2 g at 12 m	IV 10 mg/kg d1 d15 d29 and then 1/w	23.7 mg twice a day
Control	MMF 2-2.5 g/d	MMF up to 3 g or IV CYC (eurolupus)	MMF 2 g/d
GC regimen	IV pulse followed by 0.5 mg/kg starting dose 7.5 mg at 3 m	IV pulse followed by 0.5-1 mg/kg starting dose 10 mg at 6 m	IV pulse followed by 20 mg/d starting dose 2.5 at 4 m
Definition of response	UPCr <0.5 eGFR ≥85% no intercurrent event	UPCr <0.7 eGFR >80% no rescue	UPCr <0.5 eGFR >80% no rescue
Timepoint at assessment	76 w	104 w	52 w
CR at intervention arm^a^	46.4%	43%	41%
CR at control arm^a^	33.1%	32%	23%
Treatment difference	13	11	18
Primary endpoint at 6 m	NA	NA	32% vs 20%
Primary endpoint at 12 m	NA	47% vs 35%	41% vs 23%
Primary endpoint at 18 m	46.4% vs 33.1%	NA	NA
Primary endpoint at 24 m	NA	43% vs 32%	56% vs 43%
Overall response at 12 m	59.1% vs 50.7%^c^	NA	70% vs 52%^c^
Renal flares		HR 0.45 (0.28-0.72) (104 w)	OR 0.85 (0.42-1.73) b(36 m)
Renal related events or death	18.9% vs 35.6%	HR 0.51 (0.34-0.77) (104 w)	
Serious AEs	32.4% vs 18.2%	26% vs 30%	21% vs 21%

AE, adverse events; CR, complete response; eGFR, estimated glomerular filtration rate; HR, hazard ratio; m, months; NA, not available; OR, odds ratio; tbs, tablets; UPCr, urine protein to creatinine ratio; w, weeks.

a Based on each trial’s primary endpoint

b *P* = ns

c Overall response: complete and partial response.

### Biologics (belimumab, anifrolumab, and OBI)

The SLR identified 1 phase 3 RCT of low RoB (BLISS-LN) and an extension study comparing iv belimumab (BLM) with placebo, added to MMF or CYC in patients with LN.

BLISS-LN enrolled 448 patients with biopsy-proven class III/IV(±V) or V LN who were assigned 1:1 to receive iv BLM (10 mg/kg on days 1, 15, and 29 and every 28 days thereafter to week 100) or placebo, in addition to standard therapy (MMF, target dose 3 g/d, or CYC 500 mg every 2 weeks for 6 infusions followed by AZA) (Table 1, Supplementary Table S2) [20]. All patients received prednisone starting at 0.5 to 1.0 mg/kg/d tapered to ≤10 mg/d by week 24. The primary endpoint was primary efficacy renal response (PERR) (defined as UPCR ≤ 0.7, eGFR no worse than 20% below the baseline value or at least 60 mL/min/1.73 m^2^, and no use of rescue therapy for treatment failure), assessed at week 104. A significantly higher proportion of patients in the BLM group achieved PERR compared with placebo (43% vs 32%; OR 1.6, 95% CI 1.0-2.3). Complete renal response (defined as UPCR <0.5, eGFR no worse than 10% below the baseline value or ≥90 mL/min/1.73 m^2^, and no use of rescue therapy) was also more frequent in the BLM group compared with placebo (30% vs 20%; OR 1.7, 95% CI 1.1-2.7). Importantly, patients in the BLM arm had a significantly lower risk of renal flare until week 104 (HR 0.45, 95% CI 0.28-0.72) [20]. In a safety extension study of 255 patients who either remained on BLM or switched from placebo-to-BLM, PERR rates were increased in both arms 28 weeks after enrolment (placebo-to-BLM: from 60%-67%; BLM-to-BLM: from 70%-75%) (Supplementary Table S2) [21].

In addition to the BLISS-LN, the additive effect of BLM was explored in a small phase 2 RCT (RoB of some concerns) of 43 patients with class III/IV and mixed III/IV+V LN who received either iv BLM (10 mg/kg at weeks 4, 6, and 8 and monthly thereafter) or placebo on top of rituximab (RTX) (1 g), CYC (750 mg at weeks 0 and 2) and a short course of prednisone (40 mg/d as starting dose, withdrawn by week 12). At week 48, the overall response rates were similar between groups (no BLM: 41% vs BLM: 52% *P* = .45) (Supplementary Table S3) [22].

Anifrolumab (ANI) was investigated in a phase 2 RCT (RoB of some concerns), where 147 patients with class III/IV LN were randomised in 3 arms. Patients in the basic ANI regimen received 300 mg of monthly iv ANI, patients in the intensified regimen received 900 mg for the first 3 doses and 300 mg thereafter, and patients in the third arm received a placebo. MMF was used as a background treatment at 2 g/d. The primary endpoint was the change in 24 h UPCR (geometric mean ratio [GMR]; <1 favoured ANI) assessed at 52 weeks. The primary endpoint was not met (GMR = 1.03; 95% CI 0.62-1.71); however, patients in the intensified regimen had numerically higher response rates vs placebo (45.5% vs 31.1%) (Supplementary Table S2) [23].

The original SLR identified one phase 2 RCT (NOBILITY) (RoB with some concerns) that compared OBI with placebo in combination with MMF in class III/IV LN [24]. One hundred twenty-five patients were randomly assigned to OBI (1 g at weeks 0, 2, 24, and 26) or placebo in combination with MMF (2-2.5 g/d) and prednisone (0.5 mg/kg/d tapered to 7.5 mg/d by week 12). The primary endpoint was complete renal response (defined as UPCR <0.5, serum creatinine [sCr] within normal range without >15% worsening of baseline values, and inactive urinary sediment) assessed at 56 weeks; this was met in more patients in the OBI group compared with placebo (35% vs 23% respectively; %Δ 12% 95% CI −3.4% to 28%) (Supplementary Table S2). Importantly, OBI resulted in deep and sustained B cell depletion even 6 months after the last infusion (94% were depleted at week 52) [24].

### Other IS agents

One RCT of low RoB randomised 100 patients 1:1 to leflunomide (LEF) (40 mg/d for 3 days, followed by 20 mg/d for 24 weeks) or iv CYC (0.8-1.0 g monthly) in combination with a high-dose prednisone protocol (0.8-1.0 mg/kg/d tapered to 10 mg) and assessed complete and partial response after 24 weeks of treatment; no significant difference was detected (CR 23% vs 27% *P* = .64 and partial response 56% vs 42%; *P* = .16 in LEF and CYC, respectively) [25].

In a multicenter RCT (high RoB), 191 patients with LN were assigned 1:1 to a combined IS treatment with iv CYC (12 pulses 0.25-0.5 g/m^2^ at 2-week intervals for 6 months) in addition to an oral IS agent (MMF [0.75-1.00 g/d] or AZA [2 mg/kg/d] or LEF [20 mg/d]) vs iv CYC alone for 24 weeks. All patients followed a high-dose prednisone protocol. Complete remission at 24 weeks was significantly higher in the combined IS group vs CYC alone (35.8% vs 16.7%, Δ 19.1% [*P* = .003]) (Supplementary Table S3) [26].

### Glucocorticoids

Two small RCTs with similar designs and a high RoB explored the treatment difference between a high-dose prednisone regimen (1 mg/kg/d) vs a standard-dose regimen (0.5 mg/kg/d), in addition to standard treatment (MMF or CYC in one study each) (Supplementary Table S5). Notably, the treatment effect was inconsistent between studies. In the study that used CYC as background treatment (*n* = 32), there was no difference in the overall response between the high and the standard-dose glucocorticoids (GC) groups (83.3% and 86.7%, respectively) [27]. Conversely, there was a significant benefit from the high-dose GC arm in patients under MMF treatment (*n* = 20) [28]. However, the level of certainty was very low due to the small sample size and low study quality.

Following the completion of the current SLR, a phase 3 RCT with a comparable protocol to NOBILITY was published (see Methods); thus, it is presented separately. REGENCY was a phase 3 RCT of low RoB designed to test the same hypothesis as the NOBILITY trial (superiority of combination of OBI with MMF vs placebo with MMF) (Supplementary Table S2) [3]. Patients received the same dosing as in the phase 2 RCT for the first 26 weeks and then were randomly reassigned to receive an additional course of one or 2 doses of OBI between weeks 50 and 52 (3 study arms in total). The target dose of MMF was the same (2-2.5 g/d), but the GC target dose was set at 5 mg/d by week 24. The primary end point was CR at week 76 (defined as UPCR <0.5 g/g, eGFR >85% of baseline value, and no intercurrent event [ie, rescue therapy, treatment failure, death, or early trial withdrawal]). Of note, the definition of CR did not require an inactive sediment (less stringent than the endpoint of the NOBILITY). A total of 271 patients were randomised 1:1 to OBI (combined dose schedules) or placebo. At week 76, 46.4% of patients in the OBI group and 33.1% in the placebo group achieved a CR (adjusted difference 13.4 percentage points; 95% CI 2.0-24.8) [3]. When proteinuria and eGFR were analysed separately, the effect of OBI remained consistent. More OBI-treated patients had a UPCR <0.8 g/g compared with placebo (55.5% vs 41.9% adjusted difference, 13.7 percentage points; 95% CI 2.0-25.4; *P* = .02) and eGFR was increased by 2.31 ± 2.71 mL/min/1.73 m^2^ in the OBI-treated patients compared with a decrease by 1.54 ± 2.71 mL/min/1.73 m^2^ seen in the placebo group (adjusted difference, 3.84 mL/min/1.73 m^2^ 95% CI −1.83 to 9.51 at the end of follow-up).

### Subgroup and secondary analyses of RCTs

Subgroup and secondary analyses of RCTs with combination treatment arms (namely the BLISS-LN, AURORA 1, and REGENCY trials) indicated different treatment effects in different patient subgroups (Supplementary Tables S6 and S7). In particular, patients with UPCR ≥3g/g compared with those with UPCR <3g/g and patients with pure class V LN compared with those with class III/IV LN had no benefit from the addition of BLM, in terms of PERR (OR 0.85; 95% CI 0.44-0.63 and OR 0.83; 95% CI 0.27-2.62, respectively) [29]. In the AURORA trial, patients already on MMF at screening had increased odds of response (OR 5.8, 95% CI 2.8-11.9), while MMF-naive patients had no additional benefit (OR 1.3, 95% CI 0.6-2.5) [18]. In REGENCY, men were less likely to respond to combination treatment with OBI compared with women. In fact, men under placebo had higher response rates (67%) than men on OBI (43%); however, the number of men in each arm was very small [3].

Regarding treatment following initial response (formerly called ‘maintenance’ treatment), only one RCT (RoB with some concerns), including 271 patients with class III/IV, class V, or mixed class LN, compared the efficacy of LEF vs AZA in patients who had received an initial treatment with iv CYC. At 36 months, there was no difference between groups in terms of flares, time to flare, and proteinuria (Supplementary Table S4) [30].

## SAFETY

### Infections

In the aforementioned RCTs, infections were the most frequently reported adverse events (AEs), yet serious infections were far less common, ranging from 7% to 11%, except for OBI-treated patients in the REGENCY RCT (low RoB) and ANI-treated patients in TULIP-LN (RoB with some concerns) (Supplementary Table S8). Infections in the OBI arm in REGENCY were partly driven by the COVID-19 pandemic; whereas in ANI-treated patients in TULIP-LN, they were attributed to frequent herpes zoster virus infections (20.0% in the ANI basic regimen, 13.7% in the intensified regimen, and 8.2% in placebo) [3,23].

The frequency of serious infections in the placebo arms varied between 3% in BLISS-LN (low RoB) and 18% in the NOBILITY (RoB with some concerns). Interestingly, these 2 studies shared similar GC protocols and background treatments (MMF), except for a group of patients in BLISS-LN who received iv CYC instead of MMF. Although there were no striking differences between the baseline characteristics, patients in the NOBILITY trial had more adverse prognostic factors than BLISS-LN. All patients in the NOBILITY trial had class III/IV LN, with half of them having a relapsing disease; by contrast, patients in BLISS-LN had mostly new-onset LN, and class V was not excluded [20,24]. The remaining studies had less variation in the frequency of serious infections (around 10%) (Supplementary Table S3).

### Kidney function

An early transient decrease in eGFR was common in the VCS-treated patients in the AURORA 2 trial, and a similar trend (increase in sCr not exceeding 15%) was noted in the TAC-based regimen in another RCT (low RoB) (Supplementary Table S8) [16,19].

### Mortality

Deaths were infrequent and balanced between arms in all RCTs, except for the low-dose VCS group in the NOBILITY phase 2 RCT (RoB with some concerns), where mortality was higher in the low- vs high-dose VCS or placebo (11.2% vs 2.3% vs 1.1%, respectively) [17]. This finding was not confirmed in the phase 3 RCT, despite using the same low-dose VCS regimen.

### Section 3: nonimmune treatment

Only 1 phase 1 RCT of high RoB tested the efficacy and safety of SGLT2i in 28 patients with SLE, including 17 patients with LN. No safety concerns were raised; proteinuria remained stable throughout the follow-up period (24 weeks) (Supplementary Table S9). Of note, there was an increase in eGFR (7.73 mL/min/1.73 m^2^) in patients with baseline eGFR level of <90 mL/min/1.73m^2^ [31].

No new study explored the efficacy or safety of ACEi, ARBs, statins, antiosteoporotic treatments nor compared different modalities in end-stage kidney disease management.

### Section 4: treatment target

Two parts comprised this broad SLR. The first part focused on the prognostic value of commonly used endpoints, ie, CR and partial response (PR, respectively), or their individual components, at various timepoints (3, 6, 9, and 12 months). The second part explored the prognostic significance of epidemiological, clinical, and histologic factors at the time of biopsy. The outcomes of interest were long-term adverse renal events (expressed as percentage of eGFR decline, chronic kidney disease [CKD], end stage kidney disease [ESKD], relapse), and/or death.

### Prognostic value of complete and PR

Only 2 of 8 studies shared the same definition of CR assessed at the same timepoint (6 months) (Supplementary Table S10). The first study (good quality) followed 172 patients for 120 months and explored the association of CR at 6 and 12 months with long-term survival [32]. CR at 12 months but not at 6 months was significantly associated with survival rate. The second study (low quality) assessed the response status of 114 patients with LN and found that not achieving a CR at 6 months is associated with a worse prognosis (defined as failure to achieve at least PR at the end of follow-up of 28 months) [33]. Although the remaining studies (mostly of fair and good quality) had discrepancies regarding the definition of CR and/or timepoint of assessment and/or outcome of interest, the association between failure to achieve CR and adverse outcomes was significant and consistent among studies. Regarding PR, the SLR identified 7 studies (all of them, good quality) that explored the association between PR and long-term outcomes, with less variability in terms of definitions and endpoints. A consistent finding was that failure to achieve at least PR in 6 to 12 months increased the risk of CKD/ESKD at the end of follow-up (HR range 2.43-17.07) (Supplementary Table S11).

Six studies explored the association between individual components of response (proteinuria, kidney function) and long-term adverse outcomes (3 of poor [[33], [34], [35]]; 3 of good quality [[36], [37], [38]]) (Table 2) [[33], [34], [35], [36], [37], [38], [39], [40], [41]]. All but one study [33] assessed proteinuria or kidney function (measured either as sCr or eGFR) at 12 months after treatment initiation and found that patients with UPCR <0.7-0.9 g/g and patients with sCr <1-1.1 mg/dL had a lower (up to 80%) risk of developing CKD. Other components of response, namely an active urinary sediment, active serology, duration of remission, and time to remission, were occasionally reported as significant factors; however, the grade of evidence was low due to the low quality of the studies and insufficient data (Supplementary Table S12).

**Table 2:** Single predictors of long-term kidney outcomes during follow-up period (not at baseline)

Study ID	*N*	Definition		Follow-up	Outcome	Comments
		Single component				
Reference		Parameter	Timepoint of assessment			
		Duration of remission				
Zen et al 2022 [39]	270	Sustained remission for >3 y	NA	116 mo	Renal flares: OR 0.231, 95% CI: 0.058-0.920	
Gatto et al 2024 [40]	303	Clinical-SLEDAI-2K = 0 for > 1 y	NA	5 y	CKD: HR 0.830; *P* < .001	
		Time to remission				
Pirson et al 2021 [41]	103	Early remission (<7 mo) vs late (>7 mo)	7 mo		CKD: no significant difference	All patients achieved remission at various points
		Proteinuria	-			
Kapsia et al 2022 [37]	100	UPr >0.8 g/d	12 mo	72-100 mo	Flare: OR 4.12, *P* = .02 CKD >3: OR 10.8, *P* = .001	
Mackay et al 2019 [35]	550	Log proteinuria	12 mo	48 m	CKD: Log proteinuria at 12 mo HR 1.54 (95% CI 1.23-1.93) RRT: Log proteinuria at 12 mo HR 2.05 (96% CI 1.35-3.10)	1-unit increase on the natural logarithmic scale equates to an ∼2.72-fold increase on the raw scale
Braga et al 2022 [34]	214	UPr >0.9 g/24 h	12 mo	11.2 ± 7.2 y	CKD: PPV 66.0, NPV 68.8. sensitivity 63.5%, specificity 71.2% ESRD: PPV 40.8. NPV 93.2	
Moroni et al 2020 [36]	381	UPr > 1.195 g/d	12 mo	10.7 y	CKD: PPV 29, NPV 92, sensitivity = 60.7%, specificity = 75.8%	
		sCr/eGFR				
Hailu et al 2022 [33]	114	sCr	6 mo	27.93 ± 17.15 mo	No response, progression to ESRD or death: OR 0.12 (95% CI 0.030-0.475)	
Mackay et al 2019 [35]	550	Log sCr	12 mo	48 mo	CKD: Log sCr at 12 mo HR 8.84 (95% CI 4.88-16.03) RRT: Log sCr at 12 mo HR 10.16 (95% CI 4.92-20.95)	1-unit increase on the natural logarithmic scale equates to an ∼2.72-fold increase on the raw scale
Braga et al 2022 [34]	214	sCr >0.9 mg/dL	12 mo	11.2 ± 7.2 y	CKD: PPV 62.9, NPV 68.5, sensitivity 54.8%, specificity 75.3% ESRD: PPV 62.5, NPV 82.1	
Moroni et al 2020 [36]	381	sCr > 1.195 mg/dL	12 mo	10.7 y	CKD: PPV 54, NPV 91, sensitivity 48.1%, specificity 92.1%	
Farinha et al 2024 [38]	260	eGFR ≤75	12 mo	8 y	CKD: HR 22.86 (95% CI 8.38-62.36)	
		Urine sediment				
Moroni et al 2020 [36]	381	Urinary RBC >5	12 mo	10.7 y	CKD: PPV 17, NPV 92, sensitivity 61.3%, specificity 58.8%	

bsl, baseline; CI, confidence interval; CKD, chronic kidney disease serum creatinine; eGFR, estimated glomerular filtration rate; ESRD, end-stage renal disease; HR, hazard ratio; NPV, negative predictive value; OR, odds ratio; PPV, positive predictive value; RBC, red blood cells; RRT, renal replacement treatment; sCr, serum creatinine; UPr, urine protein.

### Prognostic value of baseline clinical and histological factors

Several baseline risk factors for adverse long-term outcomes were identified during the SLR, expanding the evidence base described in the SLR which informed the 2019 EULAR/ERA LN recommendations. At the time of diagnostic kidney biopsy, the strongest clinical predictors of an adverse kidney outcome were found to be impaired kidney function (23 studies), increased UPCR (17 studies), male sex (9 studies), and hypertension (12 studies). Most factors were adjusted for multiple confounders, and their effect remained consistent and significant in multivariable models. Regarding histological parameters of diagnostic kidney biopsy, the strongest predictors of CKD/ESKD were chronicity index (HR range for ESKD 1.05-1.58 and for CKD 1.18-1.32) and interstitial fibrosis/ tubular atrophy (IF/TA >25%, HR range for CKD 2.4-7.7), reported in 17 and 10 studies, respectively (Fig A, B, Supplementary Tables S13 and S14; for quality assessment of individual studies, see Supplementary Tables S18 and S19).

**Figure:**
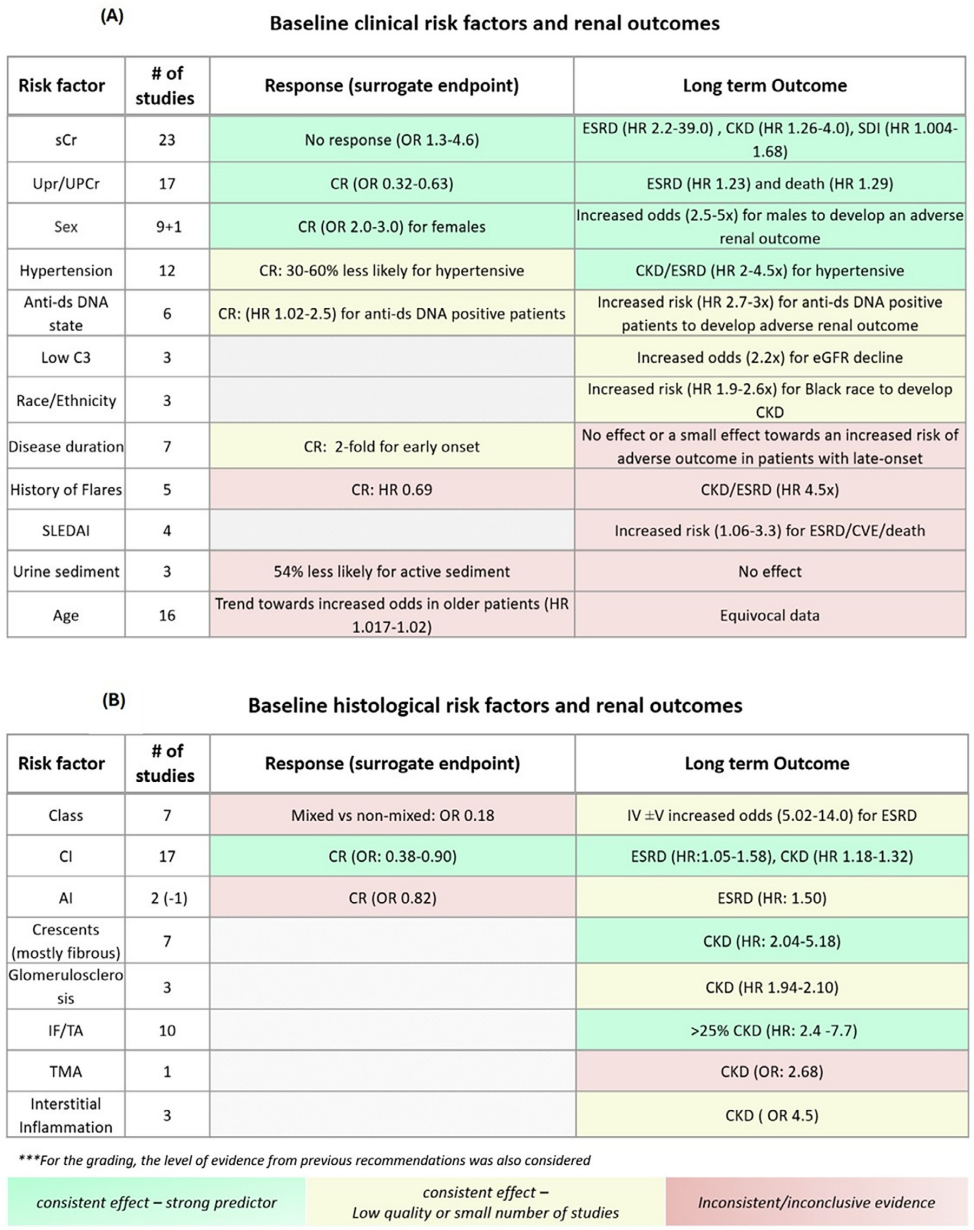
A, Association between baseline clinical factors and short- (surrogate) and long-term renal outcomes. B, Association between baseline histological factors and short- (surrogate) and long-term renal outcomes. AI, activity index; CI, chronicity index; CKD, chronic kidney disease; CR, complete response; CVE, cardiovascular event; eGFR, estimated glomerular filtration rate; ESRD, end-stage renal disease; HR, hazard ratio; IF, interstitial fibrosis; OR, odds ratio; sCr, serum creatinine; TA, tubular atrophy; TMA, thrombotic microangiopathy; UPCr, urine protein to creatinine ratio; Upr, urine protein.

### Section 5: treatment duration

This section shared the same SLR as sections 2 and 3 (Supplementary Fig S2). Two RCTs specifically in the LN population and another in patients with SLE, including LN (76% of patients), were identified (Supplementary Table S16) [[42], [43], [44]]. The WIN-Lupus investigator-initiated RCT (RoB with some concerns) was a multicenter noninferiority RCT, wherein 96 patients on sustained remission (ie, at least PR for >12 months) were randomised 1:1 to discontinue or not IS treatment. The primary endpoint was renal relapse at 24 months after discontinuation. Renal relapse occurred in 12.5% in the IS continuation group and in 27.3% in the discontinuation group, thus noninferiority was not demonstrated (difference 14.8% [95% CI −1.9 to 31.5]) [42]. The second RCT (high RoB) was a small trial including 28 patients in remission for more than a year. All patients were treated with IS plus GC for >3 years and had a repeat biopsy that showed an activity index (AI) <4. They were randomised 1:1 to discontinue glucocorticoids or IS treatment and were followed up to 12 months. Only 3 renal flares were reported, of which 2 had an AI = 2 and another had an AI = 0 in the repeat biopsy [43]. The third RCT (RoB with some concerns) followed 100 patients with SLE (76 with LN) in remission (defined as SLE Disease Activity Index [SLEDAI] <4) who were assigned to discontinue or not MMF. After 60 weeks, disease relapse (defined as an increase in SLEDAI) occurred in 8% of the MMF maintenance group vs 22% in the discontinuation group within the LN subgroup [44].

### Section 6: indications of repeat biopsy

This SLR shared the same flowchart as section 1. Eight studies presented results from patients who had a repeat biopsy, although with different indications. Four studies had patients with relapsing LN at the time of repeat biopsy [[45], [46], [47]], 2 studies had a population on stable disease or remission [13,48], and 2 studies reported on protocol biopsies [49] (Supplementary Table S17). In clinical relapse, although class switch was relatively common, ranging from 55% to 73%, a clinically meaningful change was less frequent (20%-38.4% of cases). Two studies explored the role of repeat biopsy in patients in remission or stable disease, in order to guide therapy withdrawal. In one retrospective study (fair quality), 76 patients with proliferative LN in sustained remission had a repeat kidney biopsy after ≥42 months of treatment. All patients who had histologic remission defined as an AI = 0 discontinued treatment. During a follow-up of 96 months, flares were uncommon in patients who discontinued treatment (10.9%) [48]. In another retrospective study (fair quality) of 56 patients in clinical remission, there was no difference in flares between patients with no activity and those with persistent activity (AI ≥2) at repeat kidney biopsy; however, this finding did not account for treatment continuation or withdrawal [13]. Histologic activity and chronicity in protocol biopsies were also associated with long-term outcomes. Every additional unit in the AI increased relapse risk by 20% (HR 1.2, 95% CI 1.1-1.3), whereas higher CI (>4) increased the risk of GFR decline (OR 2.9, *P* = .01) in a study (fair quality) of 42 patients followed for a median of 24.3 months after a protocol kidney biopsy [49].

## DISCUSSION

This SLR sought to provide the evidence base for the 2025 EULAR update of the management of SLE with kidney involvement. Research questions were developed to address the multifaceted clinical challenges in diagnosis, treatment, and prognosis of LN. The selection of study designs (RCTs or cohort studies) and the sample and effect sizes were largely determined by the type of each research question (prognostic, treatment, and prevention).

The SLR on diagnostic kidney biopsy and treatment targets yielded studies with consistent results; however, the quality of evidence was low to moderate. Low levels of proteinuria and/or haematuria do not preclude a histologically active disease. Performing a kidney biopsy in lower thresholds of proteinuria or in SLE cases with isolated glomerular haematuria may impact treatment management and early disease prognosis. In line with findings from the previous SLR, a strong and consistent association between CR/PR during the first 6 to 12 months and long-term outcomes was confirmed despite variations in the definition of responses [2].

The SLR on immune treatments identified high-quality yet highly heterogeneous trials. All 3 RCTs with new combination treatments (BLISS-LN, AURORA 1, and REGENCY) had similar population profiles. Patients typically had preserved kidney function (mean eGFR >90 mL/min/1.73m^2^) and high levels of proteinuria (mean UPCR ranging 3.1-4.1 g/g). Pure class V LN was excluded in the REGENCY trial and represented only a small percentage of the population in BLISS-LN and AURORA 1 (16% and 14%, respectively) [3,18,20]. All trials were successful in their primary endpoint; however, their numerical response rates were not comparable due to differences in the selection of the primary endpoint, the timeframe of assessment, the background treatment, and the GC protocol (Table 1).

Apart from the significant differences in definitions of response (including varying cutoff levels of proteinuria and eGFR assessed at different timepoints), other sources of heterogeneity may explain the (small) variations in treatment effects. The largest reported treatment difference between VCS + MMF and placebo + MMF (Δ = 18 points) was likely driven by the extremely low rates of response in the control arm (CR 23% at week 56), possibly linked to the low-dose (starting at 20 mg/d of prednisone) and rapidly tapered GC protocol and/or the low target dose of MMF (2 g/d) [18]. Likewise, the lowest treatment difference (Δ = 11 points) seen in BLISS-LN and the higher response rates in the control arm may be due to the higher GC dose (starting at 0.5-1 mg/kg) and the higher target dose of MMF (up to 3 g/d). As a low response rate in the control arm allows for a greater treatment difference, results should be interpreted with caution and studies should not be compared with each other. In addition, the management of patients with pure class V LN in different trial protocols has introduced another source of heterogeneity. The exclusion of class V from the REGENCY trial (as opposed to BLISS-LN and AURORA1) may have created an ‘advantage’ compared with other trials because patients with class V tend to respond later than patients with proliferative classes.

AEs in general, and serious infections in particular, were balanced between experimental and comparator arms within and across studies. The largest difference noted was between the OBI and placebo arms in the REGENCY trial, wherein serious infections were far more common in OBI-treated patients (16.9%) compared with placebo (7.6%). This finding was not consistent with the phase 2 NOBILITY trial, which nevertheless used OBI at lower doses. The SLR on nonimmune treatments did not identify any phase 2 or 3 RCT; thus, evidence remains at the level of the previous SLRs.

Renal relapse occurred in 25% of patients after treatment discontinuation in 2 RCTs, in which IS were completely withdrawn. Nevertheless, no strong conclusions can be drawn, as data were of moderate quality and trials were not comparable due to different designs, populations, and definitions of remission.

High-quality studies on the role of repeat kidney biopsy are lacking. Eligible studies had great heterogeneity in terms of definitions of relapse/remission/steady state, the duration of remission, and the duration/type of IS treatment. Although the role of repeat kidney biopsy has yet to be determined, several studies indicate its contribution in various states of disease.

In summary, the present SLRs provide a comprehensive overview of the current level of evidence in various aspects of LN management. Regarding immune treatment, data obtained were novel and of high quality, supporting a paradigm shift in disease management. Evidence on the role of kidney biopsy, treatment targets, and nonimmune treatments is aligned with previous SLRs, reinforcing the strength of the EULAR recommendations.

## CRediT authorship contribution statement

**Myrto Kostopoulou:** Writing – original draft, Methodology, Formal analysis, Data curation. **George Bertsias:** Supervision, Methodology. **Carlo Alberto Scire:** Writing – review & editing, Methodology. **Dimitrios T. Boumpas:** Writing – review & editing, Supervision, Project administration, Conceptualization. **Antonis Fanouriakis:** Writing – review & editing, Supervision, Methodology, Formal analysis, Data curation.

## Appendix Supplementary materials

Image, application 1

## Footnotes

Supplementary material associated with this article can be found in the online version at doi:10.1016/j.ero.2025.07.006.
