# Supplementary material for: Management of systemic lupus erythematosus with kidney involvement: systematic literature review to inform the 2025 update of EULAR recommendations
Source: EULAR Rheumatol Open. 2025 Aug 23;1(3):210–9. doi: 10.1016/j.ero.2025.07.006 (PMC13292291; doi:10.1016/j.ero.2025.07.006)

**Management of systemic lupus erythematosus with kidney involvement: Systematic literature review to inform the 2024 update of EULAR recommendations**

**Supplementary material**

**Search strategy:**

This is an overview of the search strategy and systematic literature review (SLR) methodology to inform the 2024 EULAR update for the management of systemic lupus erythematosus (SLE) with kidney involvement. The focus of this SLR was treatment of lupus nephritis (LN) (immune and non-immune) but additional topics were also considered (eg the role of kidney biopsy, treatment targets and treatment withdrawal). For each of the topics a draft of PICOs was developed based on which the corresponding search queries were formulated.

The SLR was performed in PubMed, Embase and the Cochrane Central Register of Controlled Trials published between January 2019 and March 2024. Hand searching and snowball references were also considered. For “treatment research questions” (questions 2,3 and 5) the eligibility criteria were limited to randomized controlled trials (RCTs). For prognostic/screening questions (questions 1, 4 and 6) a more inclusive strategy was considered appropriate as RCTs were lacking.

**Research questions and PICOs**

Section 1: Indications for diagnostic kidney biopsy

**Question 1: In adult patients with SLE what is the threshold of proteinuria/hematuria and/or creatinine/eGFR to perform a kidney biopsy?**

P: Patients with SLE

I(E): Various thresholds /combinations of proteinuria or/and hematuria or/and creatinine/eGFR

C: Lower levels of proteinuria/hematuria/creatinine/eGFR

O: Lupus nephritis in kidney biopsy (including classes I-VI according to the ISN/RPS classification system and other non-classified lupus-related lesions (e.g. TMA, podocytopathy)

Section 2: Treatment of lupus nephritis

**Question 2a.** **In patients with biopsy-proven, proliferative (III/IV) or mixed (III+V, IV+V) LN, what is the evidence for the efficacy and safety of therapeutic agents compared to standard of care/placebo?**

**Question 2b. In patients with biopsy-proven, non-proliferative LN, what is the evidence for the efficacy and safety of therapeutic agents compared to standard of care /placebo?**

**Question 2c. In patients with SLE and thrombotic microangiopathy in kidney biopsy what is the evidence for the efficacy and safety of therapeutic agents compared to standard of care/placebo?**

P: Patients with LN with or without thrombotic microangiopathy

I: CYC or MMF or AZA or VCS or CsA or TAC or BLM or RTX or Obi or ANI or LEF or Telitacicept or combination or multitarget or complement inhibitors or Plasmapheresis or IVIg or Steroids (including pulses IV and oral) or HCQ or antiplatelet or anticoagulant

C: soc or placebo

O: Remission or GFR or Proteinuria or relapse or CKD or ESKD or transplantation or death or infection or malignancy or steroid related AE

Section 3: Non-immune treatment of LN

**Question 3a: In patients with LN and proteinuria what is the evidence for the efficacy and safety of ARB/ACEi and SGLT2i**

P: Patients with LN and proteinuria/hypertension

I: ARB or SGLT2i or ACEi

C: placebo

O: proteinuria, eGFR, CKD, ESKD, hypertension, CVD, HbA1c, (urinary) infections, death

**Question 3b: In patients with LN and hyperlipidemia what is the evidence for the efficacy and safety of statins**

P: Patients with LN and hyperlipidemia

I: statins

C: placebo

O: CVD, CVE, death

**Question 3c: In patients with LN on steroids what is the evidence for the efficacy and safety of preventive treatment against osteoporosis**

P: Patients with LN

I: Ca or vitD or antiresorptive

C: placebo

O: fractures, osteoporosis, death

**Question 3d: In patients with LN and ESKD what is the evidence for the benefit and safety of different kidney replacement treatments?**

P: Patients with LN and ESKD

I: Replacement modality (hemodialysis/peritoneal dialysis/transplantation)

C: Other treatment/None

O: death, CVE, relapse, infections

Section 4: Treatment target

**Question 4: In patients with LN how and when should treatment response be assessed?**

P: Patients with LN

I: Treatment response (including various cut-off levels/improvement of proteinuria, hematuria, creatinine/eGFR, serum albumin, anemia, serology, extra-renal SLE activity, activity index)

C: Patients not meeting the response/remission criteria

O: CKD, ESKD, eGFR, flare, relapse

Section 5: Treatment duration

**Question 5: In patients with LN how and when should treatment be tapered and ultimately withdrawn?**

P: Patients with LN

I: withdrawal, tapering of IS, biologics or GCs or HCQ

E: response, remission, serological activity, proteinuria, eGFR, steroid exposure, histological data from repeat biopsy

C (for intervention): continuation of treatment

C (for exposure): no response/remission

O: CKD, relapse, ESKD, eGFR, steroid related AEs, damage index, infections, malignancy, death

Section 6: Repeat biopsy

**Question 6: In patients with LN when should a repeat biopsy be considered?**

P: Patients with relapsing LN or worsening kidney function/proteinuria or inadequate response or in remission prior to treatment withdrawal, or with scheduled per-protocol repeat biopsy or with quiescent LN

I: repeat kidney biopsy

C: no repeat kidney biopsy

O: relapse, remission, eGFR, CKD, ESKD, infection, malignancy, death

**Search queries:**

Dates: January 2019 to March 2024

**Medline through PubMed** “treatment questions” 2,3,5

(("Lupus Nephritis"[MeSH Terms] OR ("lupus"[All Fields] AND "nephritis"[All Fields]) OR "Lupus Nephritis"[All Fields] OR "lupus glomerulonephritis"[All Fields] OR "Lupus Nephritis"[MeSH Terms]) AND ("Random Allocation"[MeSH Terms] OR "Clinical Trial"[Publication Type] OR "Double-Blind Method"[MeSH Terms] OR "Single-Blind Method"[MeSH Terms] OR "random*"[All Fields] OR "Placebos"[MeSH Terms] OR ("placeboes"[All Fields] OR "Placebos"[MeSH Terms] OR "Placebos"[All Fields] OR "placebo"[All Fields]) OR (("ambulatory care facilities"[MeSH Terms] OR ("ambulatory"[All Fields] AND "care"[All Fields] AND "facilities"[All Fields]) OR "ambulatory care facilities"[All Fields] OR "clinic"[All Fields] OR "clinic s"[All Fields] OR "clinical"[All Fields] OR "clinically"[All Fields] OR "clinicals"[All Fields] OR "clinics"[All Fields] OR "controlled"[All Fields]) AND "trial*"[All Fields]) OR (("singl*"[All Fields] OR "doubl*"[All Fields] OR "trebl*"[All Fields] OR "tripl*"[All Fields]) AND ("blind*"[All Fields] OR "mask*"[All Fields])) OR "RCT"[All Fields] OR ("crossover"[All Fields] OR "crossovers"[All Fields]) OR ("crossing over, genetic"[MeSH Terms] OR ("crossing"[All Fields] AND "over"[All Fields] AND "genetic"[All Fields]) OR "genetic crossing over"[All Fields] OR ("cross"[All Fields] AND "over"[All Fields]) OR "cross over"[All Fields]) OR ("crossing over, genetic"[MeSH Terms] OR ("crossing"[All Fields] AND "over"[All Fields] AND "genetic"[All Fields]) OR "genetic crossing over"[All Fields] OR ("cross"[All Fields] AND "over"[All Fields]) OR "cross over"[All Fields]) OR "Treatment Switching"[All Fields] OR "Treatment Switching"[MeSH Terms] OR "RCT"[All Fields] OR "Randomized Controlled Trial"[Publication Type])) AND (2019:2024[pdat])

**Embase** “treatment questions” 2,3,5

('lupus' AND 'nephritis') OR 'lupus nephritis' OR 'lupus glomerulonephritis' OR 'lupus erythematosus nephritis'/exp) AND ('randomization'/exp OR 'clinical trial':it OR 'double blind procedure'/exp OR 'single blind procedure'/exp OR 'random*' OR 'placeboes' OR 'placebo'/exp OR 'placebos' OR 'placebo' OR (('outpatient department'/exp OR ('ambulatory' AND 'care' AND 'facilities') OR 'ambulatory care facilities' OR 'clinic' OR 'clinic s' OR 'clinical' OR 'clinically' OR 'clinicals' OR 'clinics' OR 'controlled') AND 'trial*') OR (('singl*' OR 'doubl*' OR 'trebl*' OR 'tripl*') AND ('blind*' OR 'mask*')) OR 'crossover' OR 'crossovers' OR 'crossing over'/exp OR ('crossing' AND 'over' AND 'genetic') OR 'genetic crossing over' OR ('cross' AND 'over') OR 'cross over' OR 'treatment switching' OR 'treatment switching'/exp OR 'rct' OR 'randomized controlled trial':it) AND AND ('article'/it OR 'preprint'/it)

**CENTRAL** (Cochrane's database of trials) “treatment questions” 2,3,5

#1 MeSH descriptor: [Lupus Erythematosus, Systemic] explode all trees

#2 ("systemic lupus erythematosus"):ti,ab,kw (Word variations have been searched)

#3 (lupus):ti,ab,kw (Word variations have been searched)

#4 ("glucocorticoid") (Word variations have been searched)

#5 MeSH descriptor: [Glucocorticoids] explode all trees

#6 MeSH descriptor: [Steroids] explode all trees

#7 (steroid) (Word variations have been searched)

#8 (corticosteroid) (Word variations have been searched)

#9 MeSH descriptor: [Anti-Inflammatory Agents, Non-Steroidal] explode all trees

#10 ("non-steroidal anti-inflammatory agents"):ti (Word variations have been searched)

#11 (nsaids):ti (Word variations have been searched)

#12 MeSH descriptor: [Hydroxychloroquine] explode all trees

#13 ("hydroxychloroquine") (Word variations have been searched)

#14 ("antimalarial") (Word variations have been searched)

#15 MeSH descriptor: [Quinacrine] explode all trees

#16 ("quinacrine") (Word variations have been searched)

#17 MeSH descriptor: [Methotrexate] explode all trees

#18 ("methotrexate") (Word variations have been searched)

#19 MeSH descriptor: [Leflunomide] explode all trees

#20 ("leflunomide") (Word variations have been searched)

#21 MeSH descriptor: [Calcineurin] explode all trees

#22 ("calcineurin") (Word variations have been searched)

#23 MeSH descriptor: [Cyclosporine] explode all trees

#24 ("ciclosporin") (Word variations have been searched)

#25 MeSH descriptor: [Tacrolimus] explode all trees

#26 ("tacrolimus") (Word variations have been searched)

#27 (voclosporin) (Word variations have been searched)

#28 MeSH descriptor: [Azathioprine] explode all trees

#29 ("azathioprin") (Word variations have been searched)

#30 ("azathioprine") (Word variations have been searched)

#31 MeSH descriptor: [Mycophenolic Acid] explode all trees

#32 ("mycophenolate") (Word variations have been searched)

#33 ("mycophenolic") (Word variations have been searched)

#34 MeSH descriptor: [Cyclophosphamide] explode all trees

#35 ("cyclophosphamide") (Word variations have been searched)

#36 MeSH descriptor: [Rituximab] explode all trees

#37 ("rituximab") (Word variations have been searched)

#38 (belimumab) (Word variations have been searched)

#39 MeSH descriptor: [Abatacept] explode all trees

#40 ("abatacept") (Word variations have been searched)

#41 ("biologic") (Word variations have been searched)

#42 ("intravenous immunoglobulin") (Word variations have been searched)

#43 ("plasma exchange") (Word variations have been searched)

#44 MeSH descriptor: [Plasmapheresis] explode all trees

#45 ("plasmapheresis") (Word variations have been searched)

#46 (anifrolumab) (Word variations have been searched)

#47 (obinutuzumab) (Word variations have been searched)

#48 (ofatumumab) (Word variations have been searched)

#49 (ocrelizumab) (Word variations have been searched)

#50 (atacicept) (Word variations have been searched)

#51 MeSH descriptor: [Etanercept] explode all trees

#52 ("etanercept") (Word variations have been searched)

#53 MeSH descriptor: [Adalimumab] explode all trees

#54 ("adalimumab") (Word variations have been searched)

#55 (tocilizumab) (Word variations have been searched)

#56 (secukinumab) (Word variations have been searched)

#57 (ustekinumab) (Word variations have been searched)

#58 MeSH descriptor: [Ustekinumab] explode all trees

#59 MeSH descriptor: [Interleukin 1 Receptor Antagonist Protein] explode all trees

#60 (interleukin 1 receptor antagonist) (Word variations have been searched)

#61 (anakinra) (Word variations have been searched)

#62 (tofacitinib) (Word variations have been searched)

#63 (baricitinib) (Word variations have been searched)

#64 (upadacitinib) (Word variations have been searched)

#65 (deucravacitinib) (Word variations have been searched)

#66 MeSH descriptor: [Proteasome Inhibitors] explode all trees

#67 ("protease inhibitor") (Word variations have been searched)

#68 MeSH descriptor: [Bortezomib] explode all trees

#69 ("bortezomib") (Word variations have been searched)

#70 (iberdomide) (Word variations have been searched)

#71 (litifilimab) (Word variations have been searched)

#72 MeSH descriptor: [Interleukin-2] explode all trees

#73 (interleukin 2) (Word variations have been searched)

#74 ("IL 2") (Word variations have been searched)

#75 (daratumumab) (Word variations have been searched)

#76 (CAR-T cells) (Word variations have been searched)

#77 MeSH descriptor: [Receptors, Thrombopoietin] explode all trees

#78 (romiplostim) (Word variations have been searched)

#79 (eltrombopag) (Word variations have been searched)

#80 MeSH descriptor: [Sodium-Glucose Transporter 2 Inhibitors] explode all trees

#81 (sodium glucose transporter 2 inhibitors) (Word variations have been searched)

#82 (sglt2) (Word variations have been searched)

#83 (dapagliflozin) (Word variations have been searched)

#84 MeSH descriptor: [Renin] explode all trees

#85 (renin) (Word variations have been searched)

#86 MeSH descriptor: [Angiotensins] explode all trees

#87 ("angiotensin") (Word variations have been searched)

#88 (inhibitors) (Word variations have been searched)

#89 #1 OR #2 OR #3

#90 #84 OR #85

#91 #86 OR #87

#92 #90 AND #91 AND #88

#93 #4 OR #5 OR #6 OR #7 OR #8 OR #9 OR #10 OR #11 OR #12 OR #13 OR #14 OR #15 OR #16 OR #17 OR #18 OR #19 OR #20 OR #21 OR #22 OR #23 OR #24 OR #25 OR #26 OR #27 OR #28 OR #29 OR #30 OR #31 OR #32 OR #33 OR #34 OR #35 OR #36 OR #37 OR #38 OR #39 OR #40 OR #41 OR #42 OR #43 OR #44 OR #45 OR #46 OR #47 OR #48 OR #49 OR #50 OR #51 OR #52 OR #53 OR #54 OR #55 OR #56 OR #57 OR #58 OR #59 OR #60 OR #61 OR #62 OR #63 OR #64 OR #65 OR #66 OR #67 OR #68 OR #69 OR #70 OR #71 OR #72 OR #73 OR #74 OR #75 OR #76 OR #77 OR #78 OR #79 OR #80 OR #81 OR #82 OR #83 OR #92 260458

#94 #89 AND #93 with Cochrane Library publication date Between Jan 2019 and Mar 2024, in Trials

Medline through Pubmed Questions 1 and 6

(("lupus nephritis"[Title] OR ("lupus"[Title] AND "nephritis"[Title]) OR "lupus nephritis"[Title]) AND ((("kidney"[Title] OR "kidney"[Title] OR "kidneys"[Title] OR "kidney s"[Title]) AND ("biopsie"[Title] OR "biopsy"[Title] OR "biopsy"[Title] OR "biopsied"[All Fields] OR "biopsies"[Title])) OR (("renal"[Title] OR "renals"[Title]) AND ("biopsie"[Title] OR "biopsy"[Title] OR "biopsy"[Title] OR "biopsied"[Title] OR "biopsies"[Title]))))

**Embase** Questions 1 and 6

('lupus':ti AND 'nephritis':ti OR 'lupus nephritis':ti) AND (('kidney':ti OR 'kidneys':ti OR 'kidney s':ti) AND ('biopsie':ti OR 'biopsy':ti OR 'biopsied' OR 'biopsies':ti) OR (('renal':ti OR 'renals':ti) AND ('biopsie':ti OR 'biopsy':ti OR 'biopsied':ti OR 'biopsies':ti))) AND ([article]/lim OR [article in press]/lim OR [erratum]/lim) AND [2019-2024]/py

Medline through Pubmed Question 4

("lupus nephritis"[MeSH Terms] OR ("lupus"[All Fields] AND "nephritis"[All Fields]) OR "lupus nephritis"[All Fields]) AND ("remission"[All Fields] OR "remissions"[All Fields] OR ("response"[All Fields] OR "responses"[All Fields]) OR ("proteinuria"[MeSH Terms] OR "proteinuria"[All Fields] OR "proteinurias"[All Fields]) OR ("haematuria"[All Fields] OR "hematuria"[MeSH Terms] OR "hematuria"[All Fields] OR "haematurias"[All Fields] OR "hematurias"[All Fields]) OR "serum albumin"[All Fields] OR "anemia"[All Fields] OR "serology"[All Fields] OR "activity"[All Fields]) AND 2019/01/01:2024/12/31[Date - Publication]

**Embase** Question 4

('lupus erythematosus nephritis'/de OR ('lupus' AND 'nephritis'/de) OR 'lupus nephritis'/de) AND ('remission'/de OR 'remissions' OR 'response'/de OR 'responses' OR 'proteinuria'/exp OR 'proteinuria'/de OR 'proteinurias' OR 'haematuria'/de OR 'hematuria'/exp OR 'hematuria'/de OR 'haematurias' OR 'hematurias' OR 'serum albumin'/de OR 'anemia'/de OR 'serology'/de OR 'activity'/de) AND ([article]/lim OR [article in press]/lim OR [erratum]/lim OR [letter]/lim OR [short survey]/lim OR [preprint]/lim OR ‘biopsy’/de) AND [2019-2024]/py

SLR tabulated results

**Supplementary Table 1. Studies with various indications of kidney biopsy**

| **Study ID** | **N** | **Clinical presentation** | **Biopsy result** |
| --- | --- | --- | --- |
| (Shudan Wang et al. 2022) | 151 | UPCr 0.2-0.5 g/g | 50% progressed to UPCr ≥0.5 g/g |
| (Al Arfaj and Khalil 2023) | 476 | 57.6% with UPr <0.5 g/24h  9.2% with UPr 0.5 - 1 g/24h  33.2% ≥1 g/24h | 35.9% with class III, IV, V, II/V, III/V and IV/V had UPr <0.5 g/24 h |
| (Katsuyama et al. 2020a) | 119 | Any indication of LN | Class IV 71%; III 17%; V 13% |
| (Gouda et al. 2022) | 84 | no signs of LN | Class II: 40.5% Class III: 26.2% |
| (Carlucci et al. 2022) | 54 | UPr < 1g/24h | 77.8% had class III, IV or mixed |
| (De Rosa et al. 2020) | 46 | UPr < 0.5 g/d (±hematuria/Cr≥1mg/dl) | 85% had class III or IV (±V) respectively |
| (Chedid et al. 2020) | 87 | UPr < 1g/24h | 38.6% had class III, IV or mixed |
| (Gamaleldin et al. 2022) | 40 | varied | ROC analysis to predict proliferative classes:  Proteinuria >1092 mg/d: AUC 0.806, Sens 82.61%, Spec 64.71%  stomatocytes>0.29 × 10[4]/mL: AUC 0.781, Sens 78.26%, Spec 82.35%  erythrocytes >1.4 × 10[4]/mL: AUC 0.770, Sens 86.96%, Spec 64.71% |

**Supplementary Table 2. Efficacy of immune treatments for lupus nephritis I (new agents)**

*Randomized controlled trials 2020-2024 (and their extensions)*

| **New agents** | | | | | | | | |  |
| --- | --- | --- | --- | --- | --- | --- | --- | --- | --- |
| **Study ID** | **N** | **LN class** | **Intervention** | **Comparison** | **GC** | **Duration** | **Endpoints** | **Effect size** | |
| (Rovin et al. 2019)  AURA-LV | 265 | PLF+Mx 85.3%  V 14.7% | VCS 23.7 mg  + MMF 2g/d | VCS 39.5 mg + MMF  Low-dose PBO + MMF  High-dose PBO + MMF | IV-MP  Pz 20 mg/d tapered to 2.5 mg/d at week 16 | 24 wks | CRR* | OR for low-dose VCS 2.03; 95% CI 1.01– 4.05  OR for high-dose VCS 1.59; 95% CI: 0.78–3.27 | |
| (Rovin et al. 2021)  AURORA1 | 357 | PLF+Mx 86%  V 14% | VCS 23.7 mg  + MMF 2 gr/d | PBO instead of MMF – rest as “intervention” | IV MP  Pz 20 mg/d tapered to 2.5 mg/d at wk 16 | 52 wks | CRR* | OR 2.65; 95% CI 1.64–4.27 | |
| (Saxena et al. 2024)  AURORA2 | 216 | PLF+Mx 86% V 14% | See AURORA-1 | | >75% ≤2.5 mg/day | 104 wks | CRR* (2ary) | OR 1.74; 95% CI 1.00–3.03 | |
| (Arriens et al. 2023) | 534 | PLF+Mx 86% V 14% | See original studies above | | | 48 wks | CRR* | OR 2.76; 95% CI 1.88-4.05 | |
| (R. Furie et al. 2020)  BLISS-LN | 448 | PLF+Mx 84% V 16% | BLM IV 10 mg/kg/mo + (CYC 500 mg q 2 wks for 6 infusions or MMF 3 g/d followed by AZA  or MMF resp) | PBO instead of BLM– rest as “intervention” | IV MP  Pz 0.5-1.0 mg/kg/d tapered to ≤ 10 mg/day by wk 24 | 104 wks | PERR** | OR 1.6; 95%CI, 1.0 to 2.3 | |
| (R. Furie et al. 2022) | 255 | NS | Patients on BLM continued BLM  Patients on placebo switched to BLM | | 84% receiving –mean 5-7.5 mg/d | 28 wks (after end of BLISS-LN) | PERR | PBO-to-BLM: from 60% to 67%  BLM-to-BLM: from 70% to 75% | |
| (R. A. Furie et al. 2022) NOBILITY | 125 | PLF+Mx 100% | OBI 1g on d 1 and wks 2, 24 and 26 + MMF 2-2.5 gr/d | PBO + MMF 2-2.5 gr/d | IV MP  Pz 0.5 mg/kg/d tapered to 7.5 mg/d by wk 12 | 52 wks | CRR*** | OBI: 35% - PBO:23% %Δ 12% (95% CI −3.4% - 28%) | |
| (R. A. Furie et al. 2025) REGENCY | 271 | PLF+Mx 100% | ΟΒΙ 1g on d 1 and at wks 2, 24, 26, and 52, ± wk 50+ MMF 2-2.5 gr/d | PBO + MMF 2-2.5 gr/d | IV MP  Pz 0.5 mg/kg/d tapered to 7.5 mg/d by wk 12 | 76 wks | CRR**** | OBI: 46.4% - PBO:33.1% %Δ 13.4% (95% CI 2 – 24.8%) | |
| (Jayne et al. 2022)  TULIP LN | 147 | PLF+Mx 100% | ANI BR; 300 mg or ANI IR; 900 mg for first 3 doses, 300 mg thereafter + MMF 2 g/d by wk 8 | PBO + MMF | IV MP 0.5 g x 1  Pz mean 22.3 mg/day tapered to ≤7.5 mg/d by wk 24 | 52 wks | Change in 24h UPCR (*GMR: <1 favors ANI*) | GMR 1.03 (95% CI 0.62-1.71) | |
| (Jayne et al. 2023) | 75 | PLF+Mx 100% | See TULIP-LN | | Pz ≤7.5 mg/d by wk 60  ≤5.0 mg/d by wk 80 | 104 wks | CRR (2ary) | ANI IR: 27.3% - PBO: 17.8%; Δ 9.5% (95%CI −8.4- 27.4) | |

**Supplementary Table 3. Efficacy of immune treatments for lupus nephritis (established agents)**

| **Established agents** | | | | | | | | | | | |
| --- | --- | --- | --- | --- | --- | --- | --- | --- | --- | --- | --- |
| **Study ID** | | **N** | | **LN class** | **Intervention** | **Comparison** | **GC** | **Duration** | **1ary and major endpoints** | **Result** | |
| (Atisha-Fregoso et al. 2021)  CALIBRATE | | 43 | PLF+Mx 100% | RTX 1g + IV CYC 750 mg at wks 0 and 2 +  IV BLM 10 mg/kg at wks 4, 6, and 8 and q 4 wks through wk 48 | Same without BLM | IV MP  Pz 40 mg/d tapered to 0 mg/d by wk 12 | 48 wks | CR/PR (2ary) | No BLM 41%. BLM 52% p=ns | |  |
| (Zheng et al. 2022) | | 299 | PLF+Mx 86%  V 14.0% | TAC 4 mg/d TAC trough levels of 4-10 ng/mL | IV CY 0.75 g/m2 BSA initial dose; thereafter, 0.5-1.0 g/m2 BSA q 4 wks | IV MP  Pz 0.8mg/kg/d for 4 wks tapered to 10mg/d | 24 wks | CR/PR | CR/PR: Δ 7.1%; 95%CI, −2.7%-16.9% | |  |
| (Pal et al. 2023) | | 100 | NS | TAC 0.075 mg/kg/d + AZA 2 mg/kg/d | IV CYC 0.75 q 4 wks for 6 mo | IV MP  Pz 0.5 mg/kg/d tapered to 7.5 mg/d by 3 mo | 24 wks | ORR (CR/PR) | TAC + AZA: 86.4% - IV CYC 87.7% p=ns | |  |
| (X. Zhang, Liu, and Zhang 2020) | | | 234 | PLF+Mx 70.4%  V 29.6% | MMF 1 g/d + TAC 8mg/d  (Group A) | MMF 1 g/d + CYC 600 mg x2 q 15d (group B) | IV MP  Pz 45 mg/d  Individualized tapering | 24 wks | CR/PR | Group A 90.6% Group B 80.3% (p .027) |  |
| (M. Zhang et al. 2019) | | | 100 | PLF+Mx 100% | LEF 40 mg/d for 3 d  followed by 20 mg/d | IV CYC 0.8-1.0 g monthly | Pz 0.8-1.0 mg/kg/d tapered to 10 mg/d | 24 wks | CR | CR 23% vs 27% p= 0.64 |  |
| (An et al. 2019) | | | 191 | NS | 12 CYC pulses 0.25–0.5 g/m2 of BSA at 2-w intervals for 6 mo + (MMF (0.75–1.00 g/d), AZA (2 mg/kg/d), or LEF (20mg/d)) | 12 CYC pulses 0.25–0.5 g/m2 of BSA at 2-week intervals for 6 mo | Pz 1mg/kg/d tapered to 10 mg/d | 24 w | CR | HR= 2.13, 95% CI 1.17–3.86 |  |

*UPCR ≤0·5 mg/mg, eGFR ≥ 60 mL/min or no decrease >20% from baseline, no rescue Tx, and no > 10 mg Pz/day for ≥3 days or for ≥ 7 days in total during wks 44-52

**UPCr ≤0.7, eGFR ≥ 80% pre-flare value or ≥ 60 ml/min/1.73 m2, no use of rescue Tx

**Supplementary Table 4. Efficacy of immune treatments for lupus nephritis (maintenance)**

| **Maintenance agents** | | | | | | | | |
| --- | --- | --- | --- | --- | --- | --- | --- | --- |
| **Study ID** | **N** | **LN class** | **Intervention** | **Comparison** | **GC** | **Duration** | **1ary and major endpoints** | **Result** |
| (Fu et al. 2022) | 215 | PLF+Mx: LEF 92.6% AZA 85% V: LEF 7.4% AZA 15.0% | LEF 20 mg/d | AZA 100 mg/d | Pz 10 mg/d tapered to 5 mg/d at mo 12–15 | 36 mo | Renal flares | HR 0.89, 95% CI 0.57-1.21 |
| (Mok et al. 2020) | 150 | PLF+Mx 81%  V 19% | MMF for 6 mo, then AZA | TAC, for 6 mo, then AZA | NS | 10 yrs | Renal flares | *Proteinuric flares:* 34% vs. 53% (p=0.02)  *Nephritic flares:* 37% vs 30% (p 0.36) |

**Supplementary Table 5. Efficacy of immune treatments for lupus nephritis (other agents)**

| **Other agents** | | | | | | | | |
| --- | --- | --- | --- | --- | --- | --- | --- | --- |
| **Glucocorticoid regimens** | | | | | | | | |
| **Study ID** | **N** | **LN class** | **Intervention** | **Comparison** | **GC** | **Duration** | **1ary and major endpoints** | **Result** |
| (Bandhan et al. 2022) | 32 | NS | IV CYC + Pz 1 mg/kg/d | IV CYC + Pz 0.5 mg/kg/d | Pz 1 vs. 0.5 mg/kg/d for 4 wks - tapered after 4 wks | 24 wks | CR/PR | CR: 66.7% of pts in each group (p = 0.99)  PR/CR 83.3% vs 86.7% (p =0 .99) |
| (Bharati et al. 2019) | 20 | NS | 1 mg/kg/d for 8 wks + MMF 2 g/d | 0.5 mg/kg/d for 8 wks + MMF 2 g/d | Pz reduced by 0.1 and 0.2 mg/kg/d q 4 wks in low- and standard-dose groups, respectively, to maintenance 0.1 mg/kg/d | 24 wks | CR/PR | 100% vs. 40% (p= 0.003) |
| **Hydroxychloroquine** | | | | | | | | |
| (Gheet et al. 2023) | 60 (C) | PLF+Mx 100% | HCQ 5 mg/kg/d + MMF 1200 mg/m2/d | PBO + MMF 1200 mg/m2/d | IV MP  Pz 2 mg/kg/d for 4 wks tapered to 5 mg/d by 6 mo | 52 wks | CR/PR | CR: 60% vs 40% (p=0.003)  PR: 37% vs 43% (p=0.002) |

**Supplementary Table 6. Efficacy of immune treatments for lupus nephritis II** *Results according to outcome or clinical parameter of interest (RCTs and post-hoc analyses) expressed as {OR/HR (95%CI) or Adjusted Difference (95%CI)}*

| **Study ID** | **N** | **Originator study** | **Groups** | **Proteinuria** | **EGFR and eGFR decline** | **Kidney-related event or death** | **Flares** |
| --- | --- | --- | --- | --- | --- | --- | --- |
| (Rovin et al. 2021) | 357 | AURORA-1 | VCS vs PBO | UPCR ≤ 0·5 mg/mg   HR 2.02 (1.51-2.70)  50% UPCR reduction HR 2.05 (1.62-2.60) | eGFR slope: 1.0 vs 1.1ml/min/1.73m2  30% decline in eGFR: 10% in both groups | NA | NA |
| (Saxena et al. 2024) | 216 | AURORA-2 | VCS vs PBO | UPCR ≤ 0·5 mg/mg at 36 mo: 63.6% vs 49.4%  50% UPCR reduction at 36 mo: 86.9% vs 79.3% | eGFR slope over 2 years −0.2 mL vs. −5.4 mL | NA | HR 0.85 (0.42-1.73) |
| (Arriens et al. 2023) | 534 | AURA-LV  AURORA-1 | VCS vs PBO | ≥50% reduction in UPCR: 93.7% vs 75.2%  Time to 50% UPCR reduction: HR 1.96 (1.61-2.38) | NA | NA | NA |
| (R. A. Furie et al. 2022) | 125 | *NOBILITY* | *OBI vs PBO* | UPCR <0.8 at wk 104: 71% vs 45%  %Δ 26% (9.6%-43%) | OBI: increased eGFR over 104wks:  Δ 9.7 mL/min/1.73 m2 (1.7 -18) | NA | NA |
| (Rovin et al. 2024) | 125 | NOBILITY post-hoc | OBI vs PBO | NA | 30% decline in eGFR: HR 0.20 (0.06-0.61)  eGFR slope over 1-year Δ 4.1 ml (0.14-8.08) | HR 0.40 (0.20-0.80) | HR 0.43 (0.20-0.95) |
| (R. A. Furie et al. 2025) | 271 | REGENCY | OBI vs PBO | UPCR <0.8 at week 76: OBI 55.5% vs PBO 41.9%  %Δ 13.7 (2.0-25.4) | eGFR slope over 76 wks: Δ 3.84 (−1.83 to 9.51) | NA | NA |
| (R. Furie et al. 2020) | 446 | BLISS-LN | BLM vs PBO | NA | NA | HR 0.51; (0.34- 0.77) | NA |
| (Rovin et al. 2022) | 446 | BLISS-LN secondary analysis | BLM vs PBO | NA | 30% decline in eGFR:  HR 0.47 (0.27-0.83)  eGFR slope over 1 year 3.61 ml (SE 1.76) | NA | HR 0.45 (0.28–0.72) |
| (Yu et al. 2023) | 142 | BLISS-LN (East Asian population) | BLM vs PBO | NA | NA | HR 0.37 (0.15-0.91) | HR 0.22 (0.08- 0.58) |
| (Jayne et al. 2022) | 147 | TULIP-LN | ANI vs PBO | UPCR ANI IR vs PBO (GMR=0.96; 0.55-1.69)  UPCR ≤0.7 mg/mg at wk 52  ANI IR: 50.0% vs ANI BR: 32.6% - PBO:35.6% | eGFR ≥60 or no decrease ≥20% at Wk 52: ANI IR 81.8% ANI BR 79.1% PBO 73.3% | NA | NA |
| (Mok et al. 2020) | 150 | TAC/AZA vs. MMF/AZA 10 year results | TAC vs MMF | NA | NA | NA | *Proteinuric:* TAC/AZA 34% vs. MMF/AZA 53% (p=0.02)  *Nephritic:* TAC/AZA 37% - MMF/AZA 30% (p=0.36) |

**Supplementary Table 7. Efficacy of immune treatments for lupus nephritis III**. *Results according to subgroup of interest (previous RCTs and post-hoc analyses)*

| **Study ID** | **Primary endpoint** | **Groups** | **N** | **Subgroup and results* {OR (95%CI) or Adjusted Difference (95%CI)}** | | | | |
| --- | --- | --- | --- | --- | --- | --- | --- | --- |
|  |  |  |  | ***Race*** | ***LN class*** | ***Level of baseline UPr*** | ***Concomitant therapy*** | ***New-onset LN vs. relapse*** |
| (Rovin et al. 2019)  *AURA-LV* | CRR at 48 wks | VCS vs PBO | 265 | White race OR 3.64 (1.34-9.9) | Non class V: OR 3.69 (1.83-7.44)  Class V OR 1.12 (0.21-6.05) | NA | No MMF OR 3.75 (1.66-8.46)  MMF not significant | NA |
| (Rovin et al. 2021) *AURORA-1* | CRR at 52 wks | VCS vs PBO | 357 | White race OR 1.7 (0.8-3.7) | Non-class V: OR 2.6 (1.6-4.4)  Class V: 2.7 (0.8-9.7) | NA | MMF at screening  Yes: OR 5.8 (2.8-11.9)  No: OR 1.3 (0.6-2.5) | NA |
| (Arriens et al. 2023)n*AURA-LV + AURORA-1 integrated* | CRR at 52 wks | VCS vs PBO | 534 | No race effect | Class III: OR 4.3 (1.5-11.8)  Class IV: OR 2.6 (1.5-4.4)  Class V: OR 1.5 (0.6-4.0)  Mixed III/IV+V: OR 2.6 (1.2-5.9) | UPCR<2 mg/mg: OR 3.1 (1.4-7.2)  UPCR≥2 mg/mg: OR 2.5 (1.6-3.8) | NA | NA |
| (Menn-Josephy et al. 2024) AURORA1, subgroup analysis | CRR at 52 wks | VCS vs PBO | 148 | NA | NA | UPCR≥3 g/g: OR 4.43 (1.78->9.99) | NA | NA |
| (Anders et al. 2023) post-hoc BLISS-LN | PERR at 104 wks | BLM vs PBO | 446 | NA | NA | NA | NA | *New onset*  OR 1.36 [0.85–2.20]  *Relapsing*  OR 2.31 (1.07–5.01) |
| (R. Furie et al. 2020)  BLISS-LN | PERR at 104 wks | BLM vs PBO | 446 | NA | NA | NA | MMF: OR 1.6; (1.0-2.5)  CYC: OR 1.5; (0.7-3.5) | NA |
| (Rovin et al. 2022)  BLISS-LN secondary analysis | PERR at 104 wks | BLM vs PBO | 446 | NA | Class III or IV: OR 1.82 (1.08-3.08)  Class III/IV+V: OR 1.76 (0.77-4.05)  Class V: OR 0.65 (0.23-1.86) | UPCR< 3 g/g: OR 2.44 (1.46-4.08)  UPCR≥ 3 g/g: OR 0.85 (0.44-1.63) | NA | NA |
| (Yu et al. 2023)  BLISS-LN (East Asian population) | PERR at 104 wks | BLM vs PBO | 142 | NA | Class III or IV: 3.48 (1.26-9.59)  Class III/IV+V: 0.87 (0.23-3.28)  Class V: 0.79 (0.13-4.70) | UPCR<3 g/g: OR 2.22 (0.91-5.43)  UPCR≥3 g/g: OR 1.53 (0.49-4.75) | MMF: OR 2.19 (1.01-4.74) | NA |
| (R. A. Furie et al. 2022) NOBILITY | CRR at 52 wks | OBI vs PBO | 125 | NA | NA | UPCR < 3, Δ 7.5%, p= 0.46  UPCR ≥3: Δ 21% p=0.16 | NA | NA |
| (R. A. Furie et al. 2025) | CRR at 76 wks | OBI vs PBO | 271 | Black: Δ 17.7 (−15.8-45.4)  Other: Δ 12.8 (0.7-24.9) | Mixed classes: Δ 25.5 (6.3-44.7)  Class III/IV: Δ 8.8 (−5.2-22.8) | UPCR< 3 g/g: Δ 7.8 (−7.7-23.3)  UPCR≥ 3 g/g: Δ 17.8 (1.6-33.9) | NA | New onsetΔ 23.8 (5.7-39.8), Relapsing Δ 6.0 (−9.2-21.3) |

**Supplementary Table 8. Safety of immune treatments for lupus nephritis**

| **Study ID** | **N** | **Intervention** | **Comparison** | **Infections** | **Mortality** |
| --- | --- | --- | --- | --- | --- |
| (Rovin et al. 2019)  AURA LV | 265 | VCS 23.7 mg + MMF 2g/d | VCS 39.5 mg + MMF  Low-dose PBO + MMF  High-dose PBO + MMF |  | low-dose VCS group (11.2%)  high-dose VCS (2.3%)  PBO (1.1%) |
| (Rovin et al. 2021)  AURORA1 | 357 | VCS 23.7 mg + MMF 2 gr/d | PBO instead of MMF – rest as “intervention” | VCS 65% vs. PBO 57% | VCS 1 death PBO 5 deaths |
| (Arriens et al. 2023) | 534 | AURA-LV + AURORA-1 integrated | | VCS 62.2% vs. PBO 54.9%  Serious infections: VCS 10.1% - PBO 10.2% | VCS 4.1% PBO 2.3% |
| (Saxena et al. 2024)  AURORA2 | 216 | 2-year extension of AURORA1 | | Serious infections: VCS 12.9% PBO 17.0% |  |
| (Arends et al. 2023) | 216 | Same as AURORA2 | | VCS 6% vs. PBO 12% RR 1.4 [0.97-2.06]) |  |
| (R. A. Furie et al. 2022)  NOBILITY | 125 | OBI 1g on d 1 and wks 2, 24 and 26 + MMF 2-2.5 gr/d | PBO + MMF 2-2.5 gr/d | Serious infections: OBI 8% vs. PBO 18% | OBI 1 death PBO 4 deaths |
| (R. A. Furie et al. 2025)  REGENCY | 271 | ΟΒΙ 1g on d 1 and at wks 2, 24, 26, and 52, ± wk 50+ MMF 2-2.5 gr/d | PBO + MMF 2-2.5 gr/d | Serious infections: OBI 15.4% vs PBO 6.8% | OBI 3 deaths PBO 1 death |
| (R. Furie et al. 2020)  BLISS-LN | 446 | BLM IV 10 mg/kg/mo + (CYC 500 mg q 2 wks for 6 infusions or MMF 3 g/d followed by AZA  or MMF resp) | PBO instead of BLM– rest as “intervention” | BLM 7% vs PBO 8%  Serious infections in 4% vs 3% | BLM 6 deaths PBO 5 deaths |
| (Yu et al. 2023) | 142 | Same as BLISS-LN | | BLM 10% vs PBO 16% | BLM 1 death  PBO 0 deaths |
| (Jayne et al. 2022)  TULIP-LN | 147 | ANI BR; 300 mg or ANI IR; 900 mg for first 3 doses, 300 mg thereafter + MMF 2 g/d by wk 8 | PBO + MMF | HZ: ANI BR 20%, ANI IR 13.7% PBO 8.2% | No deaths |
| (Jayne et al. 2023) | 75 | 52 wks extension of TULIP-LN | | HZ: ANI BR =1 and ANI IR =1 PBO =0 |  |
| (Atisha-Fregoso et al. 2021) CALIBRATE | 43 | RTX 1g + IV CYC 750 mg at wks 0 and 2 +  IV BLM 10 mg/kg at wks 4, 6, and 8 and q 4 wks through wk 48 | Same without BLM | Serious infections: BLM 23% vs PBO 9.5% |  |

**Supplementary Table 9. Efficacy and safety of non-immune treatments for LN**

*Only a single RCT on SGLT2i (phase 1)*

| **Study ID** | **N** | **Intervention** | **Comparison** | **Duration** | **Efficacy** | **Safety** |
| --- | --- | --- | --- | --- | --- | --- |
| (H. Wang et al. 2022) | 38 (17 with active LN) | Dapagliflozin 10 mg | None (single arm) | 24 wks | UPr: no significant change (mean at bsl 1.7gr, mean at the end 1.7g)  Mean eGFR stable for 6 months (net change of −0.24 (12.87) mL/min/1.73 m2 from baseline value of 118.40 (27.93) mL/min/1.73 m2) | 4 infections (10.5%) - 1 fungal pneumonia  0 deaths |

**Supplementary Table 10. Complete remission and long-term outcomes**

| **Study id** | **N** | **Definition** | | | **Timepoint of assessment** | **Follow-up** | **Outcome** | **Comments** |
| --- | --- | --- | --- | --- | --- | --- | --- | --- |
|  |  | **Complete remission** | | |  |  |  |  |
| **Refs** |  | Urine Protein | sCr/eGFR | Urine sediment |  |  |  |  |
| (Khosroshahi et al. 2023) | 173 | UPCr < 0.75 mg/mg | ≥60 mL/min/1.73 m^2^ or  ≤20% below the bsl value | - | 12 months | 3 years | Good kidney prognosis (eGFR >70% of bsl): OR 14.3 (95% CI 2.8–145) | Mainly Black population |
| (Cooper Blenkinsopp et al. 2022) | 173 | UPCr ≤0.7 | ≥60 mL/min/1.73 m^2^ or  ≤20% below the bsl value | - | 2 years | 5 years | ESRD or death: HR 0.33 (95% CI 0.13-0.87)  ESRD: HR 0.26 (95% CI 0.14-0.40) |  |
| (D.-J. Park et al. 2021) | 137 | if the baseline eGFR was abnormal UPCr <0.2 | eGFR> 90 mL/ min/1.73 m^2^ or a >25% increase from bsl | inactive | 12 months | 85 months | CKD: HR 0.323 (95%CI 0.153-0.685) |  |
| (K. Ichinose et al. 2019) | 172 | UPCr < 50 mg/mmol | Normal or near normal  (within 10% of a normal GFR if previously abnormal) | - | 6 and 12 months | 120 months | CR at 12 but not at 6 months was significantly correlated with the survival rate |  |
| (Hailu et al. 2022) | 114 | UPCr < 0.5 g/g from 24-h urine collection | eGFR ± 10 – 15% of bsl | - | 6 months | 28 months | poor prognosis (**n**o CR/PR at the end of follow up): OR 0.05 (95% CI 0.003–0.891) |  |
| (Enfrein et al. 2022) | 137 | UPCr<0.5 g/g (confirmed on at least two samples) | sCr<120% of baseline |  | NS | >80 months | eGFR<60: Failure to achieve CR: HR 70.60, 95% CI (14.18 to 351.45) |  |
| (Gatto et al. 2024) | 303 | UPr<0.5 g/24h >1 year | eGFR >60 ml/min per 1.73 m^2^ >1 year | - | NS | 5 years | CKD: HR 0.18 p < 0.001 |  |
| *Abbreviations: sCr serum creatinine, eGFR estimated glomerular filtration rate, UPCr Urine protein to urine creatinine ratio, bsl baseline, OR odds ratio, CI confidence interval, ESRD end stage renal disease, HR hazard ratio, CKD chronic kidney disease, CR complete remission, PR partial remission, NS not specified, UPr Urine protein* | | | | | | | | |

**Supplementary Table 11. Partial remission and long-term outcomes**

| **Study id** | N | **Definition** | | | Timepoint of assessment | Follow-up | Outcome | Comments |
| --- | --- | --- | --- | --- | --- | --- | --- | --- |
|  |  | **Partial remission** | | |  |  |  |  |
| **Refs** |  | Urine Protein | sCr/eGFR | Urine sediment |  |  |  |  |
| (D. Zhang et al. 2023) | 194 | ≥50% reduction in UPr and UPr <3 g/day | sCr ±25% or bsl improvement of sCr but not to normal range | - | 6 months | >14 months | good responders (definition based on UPr trajectory): PR at 6months OR 3.29 (95% CI 1.19-9.73), sex and, glomerulosclerosis predicted C-index 0.782 (95%CI 0.680-0.885) |  |
| (Perez-Arias et al. 2023) | 441 | ≥50% reduction in the 24h-uPCR to a value below 3 mg/mg | stable kidney function | - | 12 months | 79 months | ESRD: (vs CR) HR 2.43 (95% CI 1.18–5.00) |  |
| (Zavala-Miranda et al. 2023) | 440 | 50% reduction of the 24 h uPCR to a value below 3.0 g/g | Stable kidney function | - | NS | 79 months | decline ≥30% of eGFR, doubling of serum creatinine, ESRD: (vs CR) HR 2.59 (95% CI 1.47–4.57) | DUPLICATE WITH Perez-Arias et al |
| (Luo et al. 2022) | 107 | ≥50% reduction in UPr and UPr <3.5 g/day | normal or near normal eGFR | - | 6 months | 5 years | ESRD: Failure to achieve at least PR at 6 months HR 17.070 (95% CI 2.155-135.240) | All patients with eGFR<60ml/min/1.73m^2^ |
| (Jeon et al. 2022) | 401 | ≥50% reduction in UPr and UPr <3.5 g/day | normal or near normal eGFR | - | 6 months | 131 months | CKD: Failure to achieve at least PR at 6 months HR 2.784 (95% CI 1.606– 4.827) |  |
| (Vajgel et al. 2020) | 280 | UPr>0.3 g but <3.5 g/d or  ≥50% reduction in UPr and UPr <3.5 g/day |  | - | 12 months | 60 months | eGFR<30 ml/min/1.73m^2^ or ESKD: Failure to achieve at least PR at 12 months OR 16.3 (95% CI 3.74–71.43) |  |
| (Moroni et al. 2020) | 381 | ≥50% reduction in UPr and UPr <3.5 g/day | (near) normal eGFR | - | 12 months | 10.7 years | CKD: Failure to achieve at least PR at 12 months HR 5.165 95% CI 2.770-7.628) |  |
| *Abbreviations: sCr serum creatinine, eGFR estimated glomerular filtration rate, UPr Urine protein, bsl baseline, PR partial remission, CI confidence interval, OR odds ratio, UPCr Urine protein to urine creatinine ratio, NS not specified, ESRD end stage renal disease, HR hazard ratio,  CR complete remission, CKD chronic kidney disease* | | | | | | | | |

**Supplementary Table 12.** **Single predictors of long-term kidney outcomes during follow-up period (not at baseline)**

| **Study id** | **N** | **Definition** | | **Follow-up** | **Outcome** | **Comments** |
| --- | --- | --- | --- | --- | --- | --- |
|  |  | Single component | |  |  |  |
| **Refs** |  | Parameter | Timepoint of assessment |  |  |  |
|  |  | **Duration of remission** |  |  |  |  |
| (Zen et al. 2022) | 270 | Sustained remission for > 3 years | NA | 116 months | Renal flares: OR 0.231, 95% CI: 0.058, 0.920 |  |
| (Gatto et al. 2024) | 303 | clinical-SLEDAI-2K = 0 for > 1 year | NA | 5 years | CKD: HR 0.830; P < 0.001 |  |
|  |  | **Time to remission** |  |  |  |  |
| (Pirson et al. 2021) | 103 | early remission (less than 7 months) vs late (more than 7 months) | 7 months |  | CKD: no significant difference | All patients achieved remission at some point |
|  |  | **Proteinuria** | - |  |  |  |
| (Kapsia et al. 2022) | 100 | UPr >0.8gr/day | 12 months | 72 -100 months | Flare: OR 4.12, p = 0.02  CKD >3: OR 10.8, p = 0.001 |  |
| (Mackay et al. 2019) | 550 | Log proteinuria | 12 months | 48 months | CKD: Log proteinuria at 12 months HR 1.54 (95%CI 1.23–1.93)  RRT: Log proteinuria at 12 months HR 2.05 (96%CI 1.35–3.10) | 1-unit increase on the natural logarithmic scale equates to an ~2.72-fold increase on the raw scale |
| (Braga et al. 2022) | 214 | UPr >0.9g/24h | 12 months | 11.2 ± 7.2 years | CKD: PPV 66.0 NPV 68.8 sensitivity 63.5%, specificity 71.2%  ESRD: PPV 40.8 NPV 93.2 |  |
| (Moroni et al. 2020) | 381 | UPr > 1.195 g/day | 12 months | 10.7 years | CKD: PPV 29 NPV 92      Sensitivity= 60.7% Specificity=75.8% |  |
|  |  | **sCr/eGFR** |  |  |  |  |
| (Hailu et al. 2022) | 114 | sCr | 6 months | 27.93 ± 17.15 months | no response, progression to ESRD or death: OR 0.12 (95% CI: 0.030–0.475) |  |
| (Mackay et al. 2019) | 550 | Log sCr | 12 months | 48 months | CKD: Log sCr at 12 months HR 8.84 (95%CI 4.88–16.03)  RRT: Log sCr at 12 months HR 10.16 (95%CI 4.92–20.95) | 1-unit increase on the natural logarithmic scale equates to an ~2.72-fold increase on the raw scale |
| (Braga et al. 2022) | 214 | sCr >0.9mg/dl | 12 months | 11.2 ± 7.2 years | CKD: PPV 62.9 NPV 68.5 sensitivity 54.8%, specificity 75.3%  ESRD: PPV 62.5 NPV 82.1 |  |
| (Moroni et al. 2020) | 381 | sCr > 1.195 mg/dL | 12 months | 10.7 years | CKD: PPV 54 NPV 91      Sensitivity 48.1% Specificity 92.1% |  |
| (Farinha et al. 2024) | 260 | eGFR ≤75 | 12 months | 8 years | CKD:  HR 22.86 (95% CI 8.38–62.36) |  |
|  |  | **Urine Sediment** |  |  |  |  |
| (Moroni et al. 2020) | 381 | Urinary RBC > 5 | 12 months | 10.7 years | CKD: PPV 17 NPV 92      Sensitivity 61.3% Specificity 58.8% |  |
| *Abbreviations: OR odds ratio, CI confidence interval, CKD chronic kidney disease serum creatinine, HR hazard ratio, UPr Urine protein, RRT renal replacement treatment, PPV positive predictive value, NPV negative predictive value  sCr serum creatinine, eGFR estimated glomerular filtration rate,  bsl baseline, ESRD end stage renal disease, RBC red blood cells* | | | | | | |

**Supplementary Table 13**. Baseline clinical and epidemiological risk factors and their association with surrogate and long-term endpoints

| **Study id** | **N=** | **Risk factor** | **Follow-up** | **Surrogate** | **Outcome** |
| --- | --- | --- | --- | --- | --- |
|  |  | **Age** |  |  |  |
| (Tian et al. 2022) | 1264 | Late onset (>50yo) |  |  | ESRD: HR 1.06 (0.41, 2.74)  Death: HR 3.03 (1.39, 6.58) |
| (Mejia-Vilet et al. 2021) | 120 | Age | 2 years |  | Doubling sCr: HR 0.93, 95% CI 0.88–0.98) |
| (Mackay et al. 2019) | 550 | Age | 48 months |  | RRT: HR 0.94 (0.90–0.98) |
| (Kharouf et al. 2024) | 215 | Age | 2 years |  | Sustained ≥40% reduction in baseline eGFR: HR 0.97, 95% CI 0.94–0.99  ESRD: HR 0.92, 95% CI 0.87–0.97 |
| (Abdul Hamid et al. 2024) | 101 | Age |  | Response to treatment: OR 0.61 p=0.02 |  |
| (Gatto et al. 2024) | 303 | Age | 5 years | at least 1year of clinical-SLEDAI-2K = 0: HR: 1.017; 95% CI: 1.005–1.029 |  |
| (Jeon et al. 2024) | 296 | Age | 135.5 ± 85.9m | Relapse: by 10 years, HR 0.779, p = 0.007 |  |
| (Zoshima et al. 2024a) | 144 | Age | 72 months |  | Death: HR 3.941 95%CI 1.995–7.785 |
| (Rodelo et al. 2023a) | 285 | Age | 27 months |  | ESRD: HR 1.04, 95% CI 1.01–1.07 |
| (Perez-Arias et al. 2023) | 441 | Age | 79 months | CR: HR 1.02, 95% CI 1.01–1.03 |  |
| (Zavala-Miranda et al. 2023) * | 440 | Age | 79 months |  | >30% eGFR decline or doubling sCr, or ESRD: HR 1.15 95%CI 1.02–1.30 |
| (Frontini et al. 2022) | 187 | Age | 18.6 years |  | Death: HR 1.063, 95%CI:1.027–1.099 |
| (T. Zhang et al. 2021) | 197 | Age | 27 months |  | ESRD: HR 0.938 95%CI 0.892 0.987 |
| (Ahn et al. 2020) | 171 | Age | 57 months |  | Death: OR 1.065, 95% CI 1.018–1.114 |
| (Mackay et al. 2019) | 550 | Age | 48 months |  | RRT: HR 0.94 95%CI 0.90–0.98 |
| (Abdul Hamid et al. 2024) | 101 | Age | 6 months | CR: OR 0.612 p=0.027 |  |
| (Gatto et al. 2024) | 303 | Age | 5 years | at least 1year of clinical-SLEDAI-2K = 0: HR 1.017; 95% CI: 1.005– 1.029 |  |
|  |  |  |  |  |  |
| Refs |  | **Proteinuria** |  |  |  |
| (Kang et al. 2022) | 301 | UPr |  | CR: Significant for but Risk NA |  |
| (Katsuyama et al. 2020b) | 119 | four categories of urinary protein levels | 10 years |  | ESRD: no difference |
| (Gomez Mendez et al. 2019) | 522 | Nephrotic syndrome |  | renal response: OR 0.32, 95% CI 0.19 to 0.54 for |  |
| (Suttichet et al. 2019) | 110 | UPCr | 6 months | CR at 6m: a cut-off 4.3 had PPV 41 NPV 86 |  |
| (Kharouf et al. 2024) | 215 | UPr | 2 years |  | Death: HR 1.29, 95% CI 1.04–1.23 |
| (Zhao et al. 2024) | 122 | UPr | 12 months | CR: OR=0.63, 95% CI 0.45 - 0.89 |  |
| (Xia et al. 2024) | 98 | proteinuria ≥7.0 g/24 h | 78 months | AKI: OR 6.120 (95% CI 1.848–20.265) |  |
| (Izmirly et al. 2024) | 180 | UPCr>3 | 1 year | CR/PR: OR 3.71 95%CI 1.3–10.24 |  |
| (Chen et al. 2023) | 122 | UPr | NA |  | ESRD: HR 1.237, 95% CI: 1.025–1.491 |
| (Zoshima et al. 2024b) | 144 | UPCr<1 | 72 months |  | Death: HR 0.0160.032–0.071 |
| (Márquez-Macedo et al. 2023) | 140 | UPCr | NS |  | Recovery from RRT in patients with RRT at presentation: HR 0.91, 95% CI 0.83–0.99 |
| (Gopal et al. 2022) | 333 | UPCr | 12 months | No response: OR 1.2 95%CI 1.0–1.3 |  |
| (Perez-Arias et al. 2023) | 441 | UPr | 79 months | CR: HR 0.93, 95% CI 0.89–0.98 |  |
| (Kapsia et al. 2022) | 100 | UPr <1.5 g/day |  | shorter time to complete response: HR 1.77, p = 0.01  CR at 12m: OR 16.9, p = 0.008  CR at month 24: OR 4, p = 0.03 |  |
| (J. Zhang et al. 2021) | 376 | Tertiles of UPr | 55 months |  | All-cause mortality, and a persistent decrease in the eGFR to 50% of the baseline level: sex, class, IFTA, serum albumin, UPr  had AUC 0.82 (95% CI: 0.74–0.89) |
| (Luís et al. 2021) | 104 | UPr<2g/day | 36 months | CR: HR 1.802, 95% CI 1.161, 2.795 |  |
| (Nakano et al. 2019) | 177 | Nephrotic syndrome |  | CR: HR 0.56, 95% CI 0.39–0.81 |  |
|  |  | **sCr/eGFR** |  |  |  |
| (Luo et al. 2022) | 107 | eGFR<33ml/mi/1.73m2 | 5 years |  | ESRD: HR 3.499, 95% CI 1.044 to 11.730 |
| (Kang et al. 2022) | 301 | sCr |  | Significant for CR but RR NA |  |
| (Fan et al. 2023) | 498 | sCr | 117.5m |  | HR = 1.006; 95% CI: 1.001–1.011 for adverse renal outcomes |
| (Kharouf et al. 2024) | 215 | sCr | 2 years |  | ESRD: HR 1.01, 95% CI 1.01–1.02  Increase in SDI by ≥2: HR 1.004, 95% CI 1.00–1.01  CVE: HR 1.01, 95% CI 1.01–1.02  Death: HR 1.01, 95% CI 1.01–1.02 |
| (Zhao et al. 2024) | 122 | eGFR 30-60ml/min/1.73m^2^ | 6 months | No response: OR 4.62, 95% CI 1.02 - 20.9 |  |
| (Whittall-Garcia et al. 2024a) | 109 | sCr | 24 months | Failure to achieve CR: OR 1.33 (1.02 to 1.73) | eGFR <30 mL/min: OR 4.14 (1.19 to 14.40) |
| (Chen et al. 2023) | 122 | eGFR | NA |  | ESRD: HR 0.979, 95% CI: 0.963–0.995 |
| (Li et al. 2023) | 882 | SCr 3xtimes of baseline or sCr> 4.0 mg/dL or RRT | 53 months |  | ESRD or doubling sCr: 2.52 (95%CI 1.01–6.28) |
| (Márquez-Macedo et al. 2023) | 140 | eGFR |  |  | Recovery from RRT in patients with RRT at presentation: HR 1.29, 95% CI 1.02–1.65 |
| (Gopal et al. 2022) | 333 | sCr | 12m | No response: OR 1.9 5%CI 1.1–3.2 |  |
| (Zavala-Miranda et al. 2023) | 440 | eGFR | 79m |  | >30% eGFR decline or doubling sCr, or ESRD: HR 0.90 95%CI 0.85–0.94 |
| (Duran et al. 2022) | 116 | eGFR | NS | CR: HR 1.01 (95% CI 1.00-1.01) |  |
| (Duan et al. 2022) | 180 | sCr | 48.9 months |  | ESRD: HR 39.56, P < 0.001 |
| (Moroni, Porata, Raffiotta, Quaglini, et al. 2022) | 203 | sCr | 14 years |  | >30% eGFR decline confirmed in 3 visits for 3 months: OR, 1.68; 95% CI, 1.31 to 2.15 |
| (Jeon et al. 2022) | 401 | AKI | 131 months |  | EGFR<60ml/min/1.73m^2^: HR: 4.759; 95% CI: 2.709–8.360 |
| (Frontini et al. 2022) | 187 | AKI | 18.6 years |  | first SDI score increase: HR 1.587,95% CI:1.082–2.327 |
| (Qiu et al. 2022) | 137 | sCr | 27.2 months |  | 25% decline in eGFR or ESRD and 100% increase in the uPCR or death: OR 1.020, 95% CI 1.003–1.037 |
| (P. Liu et al. 2022) | 236 | sCr | 67.9 months |  | ESRD: HR 5.910; 95%CI 1.253–27.875 |
| (Strufaldi et al. 2021) | 253 | sCr | 30m |  | CKD or doubling sCr or death: HR 1.26 95%CI 1.09 1.45 |
| (D.-J. Park et al. 2021) | 137 | eGFR | 85 months |  | CKD: HR 0.984 (95%CI 0.972-0.997) |
| (Ahn et al. 2020) | 171 | sCr | 57 months |  | ESRD: OR 2.233, 95% CI 1.539–3.239 |
| (Saleh et al. 2020) | 100 | SCr, sCr≤1.65 | NS | CR/PR: OR 0.648 95%CI 0.49-0.855  76% sensitivity and 71% specificity |  |
| (Tselios et al. 2020) | 118 | CKD stage 4 vs CKD 3b | 10 years |  | Next stage of CKD: HR 2.76 95%CI 1.5 - 5.08 |
|  |  | **Urinary Sediment** |  |  |  |
| (Mackay et al. 2019) | 550 | Urinary RBC at bsl | 48 months |  | Not a predictor for CKD/ESRD |
| (Perez-Arias et al. 2023) | 441 | pyuria | 79m | CR: HR 1.39, 95% CI 1.01–1.91 |  |
| (Duran et al. 2022) | 116 | Active sediment | NS | CR: HR 0.46 (95% CI 0.22-0.96) |  |
|  |  | **Ethnicity/Race** |  |  |  |
| (Kharouf et al. 2024) | 215 | Race | 2 years |  | Sustained reduction ≥40% of eGFR:  White race HR 0.35, 95% CI 0.17–0.73  Chinese race HR 0.16, 95% CI 0.04–0.70 |
| (Enfrein et al. 2022) | 137 | African ethnicity | >80 months |  | eGFR<60: HR 2.63, 95% CI (1.01 to 6.89) |
| (Mackay et al. 2019) | 550 | Race |  | acute or sustained decline of ≥50% in the eGFR: HR 1.912 95%CI 1.05–3.50 |  |
|  |  | **Flare** |  |  |  |
| (Gatto et al. 2024) | 303 | Proteinuric vs nephritic | 5 years |  | eGFR<60ml/min/1.73m^2^:  70.1% less likely in proteinuric flares |
| (Liao et al. 2023) | 526 | Nephritic flares | 7.5 years |  | eGFR<15 ml/min/1.73m^2^: HR 9.39, 95% CI 2.88-30.60 |
| (Perez-Arias et al. 2023) | 441 | Second, third flare | 79 months | CR: HR 0.69, 95%CI 0.53–0.90 and HR 0.60, 95% CI 0.44–0.81, respectively |  |
| (Hailu et al. 2022) | 114 | History of flare | 28 months |  | No response, progression to ESRD or death OR 0.04; 95% CI: 0.005–0.37 |
| (Vajgel et al. 2020) | 280 | History of flare | 60 months |  | eGFR<30ml/min/1.73m^2^ or ESRD: OR 4.49; 95% CI,1.10–18.44 |
|  |  | **Duration of disease** |  |  |  |
| (Duran et al. 2022) | 116 | Newly diagnosed SLE | NS | CR: HR 2.15 (95% CI 1.26-3.67) |  |
| (Moroni, Porata, Raffiotta, Quaglini, et al. 2022) | 203 | Months from LN onset to kidney biopsy | 14 years |  | >30% eGFR decline confirmed in 3 visits for 3 months: OR, 1.01; 95% CI, 1.00 to 1.01 |
| (Jeon et al. 2022) | 401 | Delayed onset LN | 131 months |  | EGFR<60ml/min/1.73m^2^: HR: 2.934; 95% CI: 1.638–5.256 |
| (Kwon et al. 2020) | 136 | Initial onset vs early onset LN | 59.5 months | Renal flare: HR 3.12 (95%CI 1.40–6.96) | CKD: HR 4.39 (95%CI 1.07–17.96) |
| (Kunihiro Ichinose et al. 2020) | 184 | Early onset (within 5 years) vs late onset | 12 months and 123 months | CR: OR 2.10, 95% CI 1.05–4.23 | ESRD: no difference  Death: HR 0.24 95%CI 0.05–0.93 |
| (Ahn et al. 2020) | 171 | Early vs late onset | 57 months |  | ESRD: OR ns |
| (Nakano et al. 2019) | 177 | Delayed LN (>1year after SLE diagnosis) |  | CR: HR 0.48, 95% CI 0.33–0.70 |  |
|  |  | **Hypertension** |  |  |  |
| (Rong et al. 2022) |  | Hypertensive vs non-hypertensive |  |  | ESRD: HR: 1.73, 95% CI: 1.07–2.81  Doubling sCr: HR: 1.67, 95% CI: 1.10–2.53  Mortality: HR: 1.12, 95% CI: 1.15–2.57 |
| (Gatto et al. 2024) | 303 | Hypertension | 5 years | at least 1year of clinical-SLEDAI-2K = 0: HR 0.699 95% CI: 0.532–0.921 |  |
| (Hu et al. 2024) | 456 | hypertension | 78 months |  | ESKD:/ doubling sCr/>40%decline in eGFR: HR 3.786 (1.299–11.037) |
| (Zoshima et al. 2024b) | 144 | hypertension | 72 months |  | ESRD: HR 25.768 95%CI 3.278–202.534 |
| (Duran et al. 2022) | 116 | hypertension | NS | CR: HR 0.40 (95% CI 0.27-0.94) |  |
| (Duan et al. 2022) | 180 | hypertension | 48.9 months |  | Death: HR 4.93, P < 0.05 |
| (Moroni, Porata, Raffiotta, Quaglini, et al. 2022) | 203 | hypertension | 14 years |  | >30% eGFR decline confirmed in 3 visits for 3 months: OR, 4.64; 95% CI, 1.90 to 11.32 |
| (Frontini et al. 2022) | 187 | hypertension | 18.6 years |  | Death: HR 3.096, 95%CI 1.211–7.912  first SDI score increase: HR 1.669, 95%CI:1.126–2.475 |
| (Qiu et al. 2022) | 137 | hypertension | 27.2 months |  | 25% decline in eGFR or ESRD and 100% increase in the uPCR or death: OR 3.448 95%CI 1.326–8.970 |
| (Gomes et al. 2021) | 166 | hypertension | 7.7 years |  | CKD: HR 3.5 (95%CI 1.3–9.8) |
| (Reátegui-Sokolova et al. 2020) | 241 | hypertension | NS |  | Renal damage: HR=1.75 |
| (Moroni et al. 2020) | 381 | hypertension | 12 months | No response: OR. 2.567, 95% CI 1.199 to 5.884 |  |
| (Gatto et al. 2024) | 303 | Hypertension | 5 years | at least 1year of clinical-SLEDAI-2K = 0: HR 0.699; 95% CI: 0.532–0.921 |  |
|  |  | **WBC** |  |  |  |
| (Chen et al. 2023) | 122 | high neutrophil-to-lymphocyte ratio (NLR) vs. Low and medium NLR group | NA |  | ESRD: HR 3.453, 95% CI 1.260–9.464 |
| (R. Liu et al. 2023) | 388 | Eosinophils ≤0.033 × 10^9^/L | 50.8 months |  | ESRD or doubling sCr: significant factor |
| (Xue et al. 2022) | 115 | high neutrophil-lymphocyte ratio and low lymphocyte-monocyte ratio | 6m |  | >30% eGFR decline or doubling sCr or ESRD, or death: OR 1.862 95%CI 1.114 - 3.208 and OR 1.884 95%CI 1.119-3.314 respectively |
|  |  | **Serology** |  |  |  |
| (Rossi et al. 2022) | 197 | Persisted isolated low C3 6m after biopsy |  |  | ESRD or death : HR 2.46 (95% confidence interval [CI]: 1.22−4.99) |
| (Kang et al. 2022) | 301 | C3 |  | Significant for CR but RR NA |  |
| (Kharouf et al. 2024) | 215 | Low complement | 2 years |  | Sustained reduction ≥40% of eGFR:  HR 2.28, 95% CI 1.06–4.92  ESRD: HR 4.0, 95% CI 1.04–11.10 |
| (Xia et al. 2024) | 98 | serum C3 ≤0.750 g/l | 78 months | AKI: OR 6.842 (95% CI 2.221–21.071) |  |
| (Hu et al. 2024) | 152 | Complement factor H-related proteins: CFHR5>4.82 mg/L, CFHR3>86.7 mg/L | >2 years |  | Decline in eGFR, ESRD or death:  Sensitivity 85.2% Specificity 63.3% and sensitivity 85.2% specificity 60.0% respectively |
| (Tselios et al. 2020) | 118 | Positive Anti-ds DNA and low complement | 10 years |  | Next stage of CKD: HR 2.72 95%CI 1.41 5.24 |
| (Fava et al. 2024) | 103 | Anti-C1q | 1 year | CR: OR 2.22, 95% CI 1.28–4.27 |  |
| (Zhao et al. 2024) | 122 | anti-SSA/Ro60  anti-dsDNA antibody | 6 months | No response: OR 3.16, 95% CI 1.14 - 8.74 and OR 0.32, 95% CI 0.10 - 0.98 respectively |  |
| (Izmirly et al. 2024) | 180 | Positive anti-dsDNA | 1 year | CR/PR: OR 2.61 95%CI 0.93–7.33 |  |
| (Chen et al. 2023) | 122 | Positive Anti-dsDNA | NA |  | ESRD: HR 3.056, 95% CI: 1.069–8.736 |
| (Lin et al. 2023) | 388 | Positive Anti-dsDNA | 47 months |  | >30% reduction in eGFR, or ESRD or death: Significant but RR not calculated due to interaction |
| (Perez-Arias et al. 2023) | 441 | Positive Anti-dsDNA | 79 months | CR: HR 1.02, 95% CI 1.00–1.04 |  |
| (Yang et al. 2022) | 120 | Positive Anti-dsDNA | 38.5 months | Relapse: HR = 1.105, P = 0.034 |  |
|  |  | **Sex** |  |  |  |
| (Mahmood et al. 2024) | 5-11 studies | Men vs female | 1.4 to 12.2 years | OR 0.52, 95% CI: 0.39–0.68 for CR | OR 2.20 (95% CI: 1.59–3.06, I^2^ = 31.15%) for risk of doubling sCr, ESRD or eGFR<15ml/min/1.73m^2^  OR 1.50, 95% CI: 0.92–2.46 for risk of death |
| (D. Zhang et al. 2023) | 194 | Female vs male | >14 months | A set including sex (OR 3.88(0.84-28.46), new onset LN,  glomerulosclerosis, and PR within 6m predicted good responders (definition based on UPr trajectory) C-index 0.782 (95%CI 0.680-0.885) |  |
| (Kang et al. 2022) | 301 | sex |  | Significant for CR but RR NA |  |
| (Hu et al. 2024) | 456 | Male vs female | 78 months |  | ESKD:/ doubling sCr/>40%decline in eGFR: HR 4.841 (1.583–15.801) |
| (E. Park et al. 2023) | 216 | Male to female | 7.8 ± 5.11 years |  | CKD stage >3: HR 2.566; 95% CI 1.149–5.733 |
| (Gopal et al. 2022) | 333 | Male to female | 12m | No response: OR 3.9 95%CI 1.4–11.0 |  |
| (Duran et al. 2022) | 116 | Female to male | NS | CR: HR 2.15 (95% CI 1.19–3.89) |  |
| (J. Zhang et al. 2021) | 376 | Sex | 55 months |  | All-cause mortality, and a persistent decrease in the eGFR to 50% of the baseline level: sex, class, IFTA, serum albumin, UPr  had AUC 0.82 (95% CI: 0.74–0.89) |
| (Kunihiro Ichinose et al. 2020)* | 184 | Female vs male | 12 months | CR: OR 3.60, 95% CI 1.32–9.83 |  |
| (K. Ichinose et al. 2019) | 172 | Male to female | 12 months | CR: OR 0.25, 95% CI 0.09–0.67 |  |

**Supplementary Table 14. Baseline histological risk factors and their association with surrogate and long-term endpoints**

| Study id | N= | Risk factors | Follow-up | Surrogate | Outcome |
| --- | --- | --- | --- | --- | --- |
|  |  | **Class** |  |  |  |
| (Kang et al. 2022) | 301 | class |  | Significant for CR but RR NA |  |
| (Rodelo et al. 2023b) | 285 | Class IV±V | 27 months |  | ESRD: HR 5.06, 95% CI 1.82–14.09 |
| (J. Zhang et al. 2021) | 376 | Class | 55 months |  | All-cause mortality, and a persistent decrease in the eGFR to 50% of the baseline level: sex, class, IFTA, serum albumin, UPr  had AUC 0.82 (95% CI: 0.74–0.89) |
| (Okamoto et al. 2021) | 140 | Proliferative vs Membranous | 96 months |  | No difference in survival rates |
| (Kunihiro Ichinose et al. 2020) | 184 | Mixed vs non-mixed | 12 months | CR: OR 0.18, 95% CI 0.04–0.80 |  |
| (Vajgel et al. 2020) | 280 | Class IV | 60 months |  | eGFR<30ml/min/1.73m^2^ or ESRD: OR 14.91; 95% CI 1.77–125.99 |
| (Mackay et al. 2019) | 550 | Class |  | acute or sustained decline of ≥50% in the eGFR: HR not calculated due to interaction |  |
|  |  | **AI/CI** |  |  |  |
| (Mejia-Vilet et al. 2021) | 120 | CI | 2 years |  | Doubling sCr: HR 1.19, 95% CI 1.01–1.40) |
| (Kharouf et al. 2024) | 215 | CI | 2 years |  | Sustained reduction ≥40% of eGFR: HR 1.18, 95% CI 1.04–1.35  ESRD: HR 1.28, 95% CI 1.08–1.51 |
| (Abdul Hamid et al. 2024) | 101 | CI |  | Response to treatment: OR 0.38, p=0.03 |  |
| (Nunes et al. 2024) | 398 | CI, CI ≥4 | 5 years |  | RRT: independent risk factor  eGFR <60 mL/min/1.73 m^2^ sensitivity  90.2%  specificity 46.0% |
| (Izmirly et al. 2024) | 180 | CI | 1 year | CR/PR: OR 1.33 per unit decrease 95%CI 1.10–1.62 |  |
| (Zoshima et al. 2024b) | 144 | CI | 72 months |  | ESRD: HR 1.476 95%CI 1.106–1.969 |
| (Lin et al. 2023) | 296 | CI | 47 months | : | >30% reduction in eGFR, or ESRD or death: Significant but RR not calculated due to interaction |
| (Gopal et al. 2022) | 333 | CI | 12m | No response: OR 1.5 95%CI 1.2–2.0 |  |
| (Perez-Arias et al. 2023) | 441 | CI | 79 months | CR: HR 0.90, 95% CI 0.85–0.95 |  |
| (Zavala-Miranda et al. 2023) | 440 | CI | 79 months |  | >30% eGFR decline or doubling sCr, or ESRD: HR 1.08 95%CI 1.01–1.16 |
| (Duan et al. 2022) | 180 | AI | 48.9 months |  | ESRD: HR 1.50, P < 0.05 |
| (T. Zhang et al. 2021) | 197 | CI | 27 months |  | ESRD: HR 1.292 95%CI 1.029 1.622 |
| (Okamoto et al. 2021) | 140 | CI | 12 months | CR: OR 0.762, 95% CI 0.581 - 0.998 |  |
| (Umeda et al. 2020) | 170 | CI | 50.5 months |  | >30% decline in eGFR: HR 1.32 (95%CI 1.11–1.56) |
| (Kunihiro Ichinose et al. 2020) | 184 | AI | 12 months | CR: OR 0.80, 95% CI 0.68–0.94 |  |
| (Ahn et al. 2020) | 171 | CI | 57 months |  | ESRD: OR 1.475, 95% CI 1.042–2.090 |
| (Moroni et al. 2020) | 381 | CI | 12 months | No response: OR: 1.221 95% CI 1.046 - 1.419 |  |
| (Shuai Wang et al. 2020) | 283 | CI | >45 months |  | Death: RR, 1.538; 95%CI 1.294–1.828 |
| (Nakano et al. 2019) | 177 | CI>2 |  | CR: HR 0.57, 95% CI 0.33–0.99 |  |
| (K. Ichinose et al. 2019) | 172 | AI | 12 months | CR: OR OR 0.82, 95% CI 0.69–0.98 |  |
|  |  | **Glomerulosclerosis** |  |  |  |
| (D. Zhang et al. 2023) | 194 | Glomerulosclerosis | >14 months | A nomogram, composed of sex/female (OR 3.88(0.84-28.46) new onset LN glomerulosclerosis and PR within 6m predicted good responders (definition based on UPr trajectory) C-index 0.782 (95%CI 0.680-0.885 |  |
| (Moroni, Porata, Raffiotta, Quaglini, et al. 2022) | 203 | glomerulosclerosis | 14 years |  | >30% eGFR decline confirmed in 3 visits for 3 months: OR, 2.12; 95% CI, 1.00 to 4.50 |
| (Umeda et al. 2020) | 170 | glomerulosclerosis | 50.5 months |  | >30% decline in eGFR: HR 1.94, 95% CI 1.11–3.39) |
|  |  | **Crescents** |  |  |  |
| (Luo et al. 2022) | 107 | fibrous | 5 years |  | ESRD: HR 3.439, 95% CI 1.029 to 11.490 |
| (Liao et al. 2023) | 526 | Cellular crescents for males  Fibrous crescents for females | 7.5 years |  | Mortality:  HR 1.91, 95% CI 1.02-3.57 and HR 5.70, 95% CI 1.61-20.25 respectively |
| (Lin et al. 2023) | 296 | crescents | 47 months |  | >30% reduction in eGFR, or ESRD or death:  Significant but RR not calculated due to interaction |
| (Moroni, Porata, Raffiotta, Quaglini, et al. 2022) | 203 | Fibrous crescents | 14 years |  | >30% eGFR decline confirmed in 3 visits for 3 months: OR, 5.18; 95% CI, 2.43 to 11.04 |
| (Strufaldi et al. 2021) | 253 | Crescents | 30m |  | CKD or doubling sCr or death: HR 3.03 95%CI 1.50- 6.10 |
| (Umeda et al. 2020) | 170 | Fibrous crescents | 50.5 months |  | >30% decline in eGFR: HR 2.04, 95% CI 1.03–4.03 |
| (Tao et al. 2020) | 101 | Fibrous crescents | 128 months |  | >30% decline eGFR or ESRD or death: HR 4.100 95% CI 1.544–10.890 |
|  |  | **IF/TA** |  |  |  |
| (Kang et al. 2022) | 301 | TA |  | Significant for CR but RR NA |  |
| (Leatherwood et al. 2019) | 202 | moderate/severe IFTA vs. none/mild |  |  | ESRD: HRSD 5.18, 95% CI 2.53, 10.59 and increased to HRSD 6.75, 95% CI 2.90, 15.70 in proliferative classes  Death: HR 3.85, 95% CI 1.07, 13.90 |
| (Hu et al. 2024) | 456 | Severe (>50%) IF | 78 months |  | ESKD:/ doubling sCr/>40%decline in eGFR: HR 3.729 (1.328–10.468) |
| (Liao et al. 2023) | 526 | TA | 7.5 years |  | eGFR<15 ml/min/1.73m^2^: HR 1.75, 95% CI 1.26-2.44 |
| (Kapsia et al. 2022) | 100 | IF/TA>25% | 100 months |  | CKD stage>3: OR 7.7, p = 0.01 |
| (J. Zhang et al. 2021) | 376 | IF/TA | 55 months |  | All-cause mortality, and a persistent decrease in the eGFR to 50% of the baseline level: sex, class, IFTA, serum albumin, UPr  had AUC 0.82 (95% CI: 0.74–0.89) |
| (Gomes et al. 2021) | 166 | IF/TA moderate/severe vs. mild/absent | 7.7 years |  | CKD: HR 2.4 (95%CI 0.9–6.7) |
| (Umeda et al. 2020) | 170 | TA, IF | 50.5 months |  | >30% decline in eGFR: HR 1.68, 95% CI 1.04–2.71 and HR 2.01, 95% CI 1.27–3.20 respectively |
| (Tao et al. 2020) | 101 | IF/TA  25–50%, IF/TA>50% | 128 months |  | >30% decline eGFR or ESRD or death: HR 3.774 95% CI 1.155–12.337 and HR 8.584 95% CI 2.509–29.367 respectively |
| (Vajgel et al. 2020) | 280 | IF > 25% | 60 months |  | eGFR<30ml/min/1.73m^2^ or ESRD: OR 5.87; 95% CI, 1.32–26.16 |
|  |  | **Vascular injury** |  |  |  |
| (Leatherwood et al. 2019) | 202 | moderate/severe vascular injury vs. none/mild |  |  | ESRD: HRSD 2.13, 95% CI 1.21 but not significant when further adjusted for class  Death: HR 2.68, 95% CI 0.98, 7.28 |
| (Strufaldi et al. 2021) | 253 | TMA | 30m |  | CKD or doubling sCr or death: HR 2.68 95%CI 1.42 - 5.03 |
|  |  | **Interstitial Inflammation** |  |  |  |
| (J. Wang et al. 2024) | 430 | High levels of macrophages CD68+ in the interstitium | NA | Treatment response:  HR 0.451; 95% CI, 0.231, 0.878 |  |
| (Liao et al. 2023) | 526 | Interstitial inflammation | 7.5 years |  | eGFR<15 ml/min/1.73m^2^: HR 4.55, 95% CI 1.88-11.00 |
| (Duong et al. 2023) | 125 | total cortical interstitial inflammation (scarred and unscarred), NIH interstitial inflammation (only in unscarred parenchyma) | 35.7 months |  | >30% reduction of eGFR: HR 2.45 (95% CI 1.2–4.97) and not significant respectively |
| (T. Zhang et al. 2021) | 197 | Tubulointerstitial CD8+T cells >130/mm^2^ | 27 months |  | ESRD: HR 1.007; 95% CI 1.003 to 1.011  85.0% sensitivity and 71.8% specificity |
| (Gomes et al. 2021) | 166 | Tubulointerstitial inflammation moderate/severe vs mild/absent | 7.7 years |  | CKD: HR 4.9 (95%CI 1.4–17.3) |
|  |  | **Immunofluorescence/Immunohistochemistry** |  |  |  |
| (Fan et al. 2023) | 498 | scanty immunodeposits vs typical ID | 117.5m |  | no difference in renal and patient survival |
| (Zavala-Miranda et al. 2023) | 105 | exostosin-1/exostosin-2 (EXO-1/2) positive immunohistochemistry | 100 months |  | progression to a 40% decline of the eGFR: lower rates  Doubling sCr/ESRD: no difference |
| (Yang et al. 2022) | 120 | C4d deposition | 38.5 months | Relapse: HR = 1.007, P = 0.040 |  |

**Supplementary Table 15.** Baseline biomarkers and their association with surrogate and long-term endpoints

| Study id | N= | Risk factor | Follow-up | Surrogate | Outcome |
| --- | --- | --- | --- | --- | --- |
|  |  | **Biomarkers** |  |  |  |
| (Mejia-Vilet et al. 2021) | 120 | urinary epidermal growth factor (EGF) | 2 years |  | Doubling sCr: urinary **EGF/Cr** levels (HR 0.88, 95% CI 0.77–0.99) |
| (Fava et al. 2024) | 127 | urine proteomic profiles (1,200 proteins) |  |  |  |
| (Gao et al. 2020) | 6 studies | uNGAL levels |  | sensitivity, 0.80 (0.57-0.92); specificity, 0.67 (0.58-0.75); AUC value, 0.74 (0.70-0.78) for renal relapse |  |
| (Suttichet et al. 2019) | 110 | High urine TWEAK levels at 3 months | 6 months | OR 0.39 (95% CI 0.17 to 0.92) for CR |  |
| (Whittall-Garcia et al. 2024b) | 109 | NET remnants (Elastase-DNA and HMGB1-DNA) | 24 months | Failure to achieve CR: Elastase-DNA: OR 2.34 (1.24–4.38) and HMGB1-DNA: OR 2.61 (1.19–5.96) | decline in eGFR ≥30%: Elastase-DNA: OR 1.55 (1.16 to 2.08), HMGB1-DNA: OR 1.54 (1.04–2.34)  and  eGFR <30 mL/min: Elastase-DNA: OR 2.84 (1.34 to 6.04), HMGB1-DNA: OR 2.04 (1.12-3.72) |
| (Ayoub et al. 2022) | 246 | Thirteen urine proteins | 12m | uPCR < 0.8 AND (eGFR > 90 ml/min at 1 year OR eGFR showed no more than a 25% reduction from baseline): AUC 0.74 (0.67, 0.82) for a model including UPCr, eGFR, C4, age, race, adiponectin, and EGF and TIM1/KIM1 |  |
| (Wu et al. 2021) | 116 | miR-485-5p levels in serum | 5 years |  | ESRD: HR 3.438; 95% CI] 1.149-10.287 |

**Supplementary Table 16.** Treatment tapering and withdrawal

| **Study ID** | **N** | **Intervention** | **Disease state at withdrawal** | **Duration of state before withdrawal** | **IS before withdrawal** | **GC at withdrawal** | 1. **Outcome** 2. **Subgroup or prognostic factor** |
| --- | --- | --- | --- | --- | --- | --- | --- |
| (Jourde-Chiche et al. 2022) | 96 | 48 d/c IS - 48 on IS | CR or PR + inactive sediment + normal or stable GFR | 12 mo | AZA or MMF for 2-3 years | ≤10 mg/d | Relapse Δ 14.8 (−1.9 to 31.5)- Non-inferiority not shown |
| (Chakravarty et al. 2024) | 100 (76% LN) | 51 d/c MMF - 49 continued MMF | SLEDAI < 4 | NS | MMF stable or decreasing for ≥2 years |  | LN group: 8% in maintenance group vs. 22% in d/c group had a relapse by 60 wks (SLEDAI increase) |
| (Gopal et al. 2022) | 28 - 2nd bx prior Tx d/c - Pts with AI < 4/24 randomized | 15 Pz d/c - 13 IS d/c | Clinical remission | ≥ 1 year | AZA/MMF/MTX/CsA/TAC for ≥ 3 years | ≤7.5 mg/d | 3/28 had renal flares (one had AI 0 in bx 2, two had AI>0) |
| (Panagiotopoulos et al. 2024) | 111 | Tapering IS until d/c |  | D/c at median 57 mo after renal response |  |  | **17.1% flared** during IS tapering (11 renal - 9 extra-renal)  **Longer time to achieve CR** [OR 1.07, P 0.046] and **higher SLEDAI-2K at tapering initiatio**n (OR: 2.57, P 0.008) associated with higher risk of renal flares during tapering  HCQ use (OR: 0.28, P 0.08) and lower SLEDAI-2K 12 months before IS d/c (OR: 1.70, P 0.013) decreased risk of post-D/C flares |

**Supplementary Table 17. Indications of repeat biopsy**

| **Repeat biopsy to guide therapy** | | | | | |  |
| --- | --- | --- | --- | --- | --- | --- |
| **Study ID** | **N with repeat bx** | **State of LN at repeat biopsy** | **Time after bx #1** | **Treatment change after bx #2** | **Outcomes (relapses, ESKD, response) and prognostic factors in repeat biopsy** |  |
| (Malvar et al. 2020) | 76 | Remission or stable state (CR ≥12 mo) | ≥42 mo of Tx | D/c IS (n=55 with AI =0 on repeat bx) -Maintenance IS (21 with AI>0) | 6/55 (9%) from the d/c group flared vs. 1/21 (4.5%) who remained on IS  *No control arm, but rate of relapse lower than rates reported in the literature (without repeat kidney bx)* |  |
| (Das et al. 2021) | 29 | NIH AI=0 on repeat bx vs. AI>0 | Mean 68 mo |  | In "sustained remission for > 48-months" group, 100% achieved histologic remission, vs. 84% in 24–48-months group (n=2 pts) |  |
| (Lledó-Ibáñez et al. 2022) | 56 | Clinical remission (CRR in 51/56) | Median 41 mo | 62.5% d/c IS - 37.5% maintained IS | Renal flare in 9/35 who d/c IS (25.7%) vs. 9/21 who continued IS (43%) |  |
| **Repeat biopsy as predictor for long-term outcomes** | | | | | |  |
| (Gatto et al. 2022) | 92 | Flare 80.5%, refractory 19.5% | NS | NS | Predictors of ESKD at bx #2: AI: HR 95% CI 1.20 (1.03 -1.41), CI: HR 1.41 (1.09 -1.82), 24h-UPr 1.22 (1.04 to 1.42)  **No histological predictors at 1st biopsy (95% CI)* |  |
| (Moroni, Porata, Raffiotta, Frontini, et al. 2022) | 61 | Flare 77%- protocol bx 23% | 49 mo | NS | Predictors of ≥30% GFR decrease at last f-u at bx #2: AI >3 (OR: 3.23; p=0.013) and CI>4 (OR: 2.90; p 0.01)  **No histological predictors at 1st biopsy* |  |
| (Gupta et al. 2020) | 62 | Flare 56% - Refractory 44% | Median 25 mo | NS | Predictors of response at 12 mo after bx #2: IFTA >30% OR: 0.12; (0.02–0.7) and TMA OR: 0.17 (0.03–0.98) |  |
| (Parodis et al. 2020) | 42 | Protocol bx | Median 24.3 mo | NS | 10/42 (23.8%) with UPCR<1.0 g/g still had AI>3 at repeat bx  Predictors of relapse at bx #2: AI (continuous)(HR 1.2; (1.1, 1.3)  Predictors of sustained SCr increase 120% of baseline at bx #2: i) CI (continuous) (HR 1.8,(1.1, 2.9), ii) interstitial inflammation, iii) IFTA  **No histological predictors at 1st biopsy* |  |

**Risk of Bias assessment**

**Supplementary Table 18.** NOS scale assessment tool for cohort and case control studies

|  | Exposed cohort representative | Selection of non-exposed cohort | Ascertainment of exposure | Outcome not present at start | Based on design or analysis | Assessment of outcome | Timing of follow-up | Adequate follow-up | Quality of study |
| --- | --- | --- | --- | --- | --- | --- | --- | --- | --- |
|  |  |  |  |  |  |  |  |  |  |
| Fava et al 2024 38258904 |  |  | ☆ | ☆ |  | ☆ | ☆ | ☆ | poor |
| Zhang et al 2023  37208030 |  | ☆ | ☆ | ☆ | ☆☆ | ☆ | ☆ | ☆ | good |
| Luo et al 2024  36581380 |  | ☆ | ☆ | ☆ | ☆☆ | ☆ | ☆ | ☆ | good |
| Rossi et al 2022  36506236 | ☆ | ☆ | ☆ | ☆ | ☆☆ | ☆ | ☆ | ☆ | good |
| Khosroshahi et al 2023  36121035 | ☆ |  | ☆ |  | ☆ | ☆ | ☆ |  | fair |
| Kang et al 2022  35764016 |  | ☆ | ☆ | ☆ | ☆☆ | ☆ | ☆ | ☆ | good |
| Tian et al 2022  35712095 | ☆ | ☆ | ☆ | ☆ | ☆ | ☆ | ☆ | ☆ | good |
| McDonald et al 2022  35640982 | ☆ | ☆ | ☆ | ☆ | ☆☆ | ☆ |  | ☆ | good |
| Rong et al 2022 33686211 | ☆ | ☆ | ☆ | ☆ | ☆ | ☆ |  |  | poor |
| Mejia-Vilet et al 2021 32892508 | ☆ | ☆ | ☆ | ☆ | ☆☆ | ☆ | ☆ |  | good |
| Almaani et al 2020  32775830 | ☆ | ☆ | ☆ | ☆ |  | ☆ |  | ☆ | poor |
| Katsuyama et al 2020  32487161 |  | ☆ | ☆ | ☆ |  | ☆ |  |  | poor |
| Gomez Mendez et al 2019  31080631 | ☆ | ☆ | ☆ | ☆ |  | ☆ | ☆ |  | poor |
| Mackay et al 2019  30225865 | ☆ | ☆ | ☆ | ☆ | ☆☆ | ☆ |  |  | poor |
| Blenkinsopp et al 2021  L2020161917 | ☆ | ☆ | ☆ | ☆ | ☆ |  | ☆ | ☆ | good |
| Suttichet et al 2019  L627651871 |  | ☆ | ☆ | ☆ | ☆☆ | ☆ |  | ☆ | good |
| Fan et al 2023  37341964 |  | ☆ | ☆ | ☆ | ☆☆ | ☆ | ☆ | ☆ | good |
| Leatherwood et al 2019  31277928 | ☆ | ☆ | ☆ | ☆ | ☆☆ | ☆ |  |  | poor |
| Kharouf et al 2024  39133193 | ☆ | ☆ | ☆ | ☆ | ☆☆ | ☆ | ☆ | ☆ | good |
| Abdul Hamid et al 2024  39002996 |  |  | ☆ | ☆ | ☆ | ☆ |  | ☆ | fair |
| Fava et al 2024  38962936 | ☆ | ☆ | ☆ | ☆ | ☆☆ | ☆ | ☆ | ☆ | good |
| Zavala-Miranda et al 2024  38922553 |  | ☆ | ☆ | ☆ |  | ☆ | ☆ |  | poor |
| Gouda et al 2022 36133925 |  |  | ☆ | ☆ | ☆ | ☆ |  | ☆ | fair |
| Carlucci et al 2022 35212719 | ☆ | ☆ | ☆ | ☆ | ☆ | ☆ | ☆ | ☆ | good |
| Gamaleldin et al |  |  | ☆ | ☆ |  | ☆ | ☆ |  | poor |
| Chedid et al 2020 33305127 | ☆ | ☆ | ☆ | ☆ | ☆☆ | ☆ | ☆ | ☆ | good |
| Al Arfaj AS et al 2023 |  |  | ☆ | ☆ |  | ☆ | ☆ |  | poor |
| Zhao et al 2024  38806217 |  | ☆ | ☆ | ☆ | ☆☆ | ☆ |  |  | poor |
| Gatto et al 2024  38765576 | ☆ | ☆ | ☆ | ☆ | ☆☆ | ☆ | ☆ | ☆ | good |
| Xia et al 2024  38835511 |  | ☆ | ☆ | ☆ | ☆☆ | ☆ | ☆ |  | good |
| Farinha et al 2024  38648778 | ☆ | ☆ | ☆ | ☆ | ☆ | ☆ | ☆ | ☆ | good |
| Nunes et al 2024  38514381 |  | ☆ | ☆ | ☆ |  | ☆ | ☆ |  | poor |
| Huang et al 2024  38485471 |  | ☆ | ☆ | ☆ |  | ☆ | ☆ | ☆ | poor |
| Aliyi et al 2024  38454130 |  | ☆ | ☆ | ☆ | ☆ | ☆ |  |  | poor |
| Wang et al 2024  38415246 |  | ☆ | ☆ | ☆ | ☆☆ | ☆ | ☆ |  | good |
| Izmirly et al 2024  38378664 | ☆ | ☆ | ☆ | ☆ | ☆ | ☆ |  |  | poor |
| Jeon et al 2024  38247126 |  | ☆ | ☆ | ☆ | ☆ | ☆ | ☆ |  | good |
| Whittall-Garcia et al 2024  38177067 |  | ☆ | ☆ | ☆ | ☆ | ☆ | ☆ | ☆ | good |
| Hu et al 2024  38158842 | ☆ | ☆ | ☆ | ☆ | ☆☆ | ☆ | ☆ |  | good |
| Park et al 2023  38148127 |  | ☆ | ☆ | ☆ | ☆☆ | ☆ | ☆ |  | good |
| Chen et al 2023  38125645 |  | ☆ | ☆ | ☆ | ☆ |  |  |  | poor |
| Zoshima et al 2023  38001036 |  | ☆ | ☆ | ☆ | ☆☆ | ☆ | ☆ | ☆ | good |
| Li et al 2023  37708856 | ☆ | ☆ | ☆ | ☆ | ☆☆ |  | ☆ |  | poor |
| Panagiotopoulos et al 2023  37308249 |  | ☆ | ☆ | ☆ | ☆ | ☆ | ☆ | ☆ | good |
| Márquez-Macedo et al 2023  37188962 |  | ☆ | ☆ | ☆ | ☆ | ☆ | ☆ |  | good |
| Liao et al 2023  37003606 |  | ☆ | ☆ | ☆ | ☆☆ | ☆ | ☆ |  | good |
| Lin et al 2023  36732805 | ☆ | ☆ | ☆ | ☆ | ☆☆ | ☆ | ☆ | ☆ | good |
| Liu et al 2023  36657400 | ☆ | ☆ | ☆ | ☆ |  | ☆ | ☆ |  | poor |
| Rodelo et al 2023  36647707 |  | ☆ | ☆ | ☆ | ☆☆ | ☆ |  |  | poor |
| Gopal et al 2022  36553169 | ☆ | ☆ | ☆ | ☆ | ☆☆ | ☆ |  |  | poor |
| Perez-Arias et al 2022  36318456 | ☆ | ☆ | ☆ | ☆ | ☆☆ | ☆ | ☆ |  | good |
| Enfrein et al 2022  36283757 |  | ☆ | ☆ | ☆ | ☆ | ☆ |  |  | poor |
| Duong et al 2022  36220148 | ☆ | ☆ | ☆ | ☆ | ☆☆ | ☆ | ☆ |  | good |
| Kapsia et al 2022  36078950 |  | ☆ | ☆ | ☆ | ☆☆ | ☆ | ☆ |  | good |
| Blenkinsopp et al 2022  36038160 | ☆ | ☆ | ☆ | ☆ | ☆☆ | ☆ | ☆ | ☆ | good |
| Zavala-Miranda et al 2023  35822600 | ☆ | ☆ | ☆ | ☆ | ☆☆ | ☆ | ☆ |  | good |
| Hailu et al 2022  35715762 |  | ☆ | ☆ | ☆ | ☆ | ☆ |  |  | poor |
| Duran et al 2022  35658643 |  | ☆ | ☆ | ☆ | ☆☆ | ☆ | ☆ |  | good |
| Duan et al 2022  35435603 |  | ☆ | ☆ | ☆ | ☆ |  | ☆ |  | poor |
| Moroni et al 2021  35368572 | ☆ | ☆ | ☆ | ☆ | ☆☆ | ☆ | ☆ |  | good |
| Jeon et al 2022  35178647 | ☆ | ☆ | ☆ | ☆ | ☆☆ | ☆ | ☆ |  | good |
| Xue et al 2022  35173851  CASE CONTROL |  | ☆ |  | ☆ |  |  | ☆ |  | poor |
| Braga et al 2022  34983697 | ☆ | ☆ | ☆ | ☆ |  | ☆ | ☆ |  | poor |
| Ayoub et al 2022  34871620 | ☆ | ☆ | ☆ | ☆ | ☆ | ☆ | ☆ |  | good |
| Frontini et al 2021  34494962 |  | ☆ | ☆ | ☆ | ☆☆ | ☆ | ☆ | ☆ | good |
| Pirson et al 2021  34446568 |  | ☆ | ☆ | ☆ |  | ☆ | ☆ |  | poor |
| Qiu et al 2022  34379208 |  | ☆ | ☆ | ☆ | ☆☆ | ☆ |  |  | poor |
| Zhang et al 2021  34217995 |  | ☆ | ☆ | ☆ | ☆☆ | ☆ | ☆ |  | good |
| Zhang et al 2021  33954909 | ☆ | ☆ | ☆ | ☆ | ☆☆ | ☆ | ☆ |  | good |
| Zen et al 2022  33909900 | ☆ | ☆ | ☆ | ☆ | ☆☆ | ☆ | ☆ |  | good |
| Liu et al 2022  33909520 |  | ☆ | ☆ | ☆ | ☆☆ | ☆ | ☆ |  | good |
| Wu et al 2021  33742995 |  | ☆ | ☆ | ☆ | ☆☆ | ☆ | ☆ |  | good |
| Gomes et al 2021  33721269 |  | ☆ | ☆ | ☆ |  | ☆ | ☆ |  | poor |
| Strufaldi et al 2021  33570723 |  | ☆ | ☆ | ☆ | ☆☆ | ☆ |  |  | poor |
| Luis et al 2021  33560332 | ☆ | ☆ | ☆ | ☆ | ☆☆ | ☆ | ☆ |  | good |
| Wang et al 2021  33558437 |  | ☆ | ☆ | ☆ | ☆☆ | ☆ | ☆ |  | good |
| Reátegui-Sokolova et al 2020  33310863 | ☆ | ☆ | ☆ | ☆ | ☆☆ | ☆ | ☆ |  | good |
| Okamoto et al 2021  33166998 |  | ☆ | ☆ | ☆ | ☆☆ | ☆ | ☆ |  | good |
| Umeda et al 2020  33148339 |  | ☆ | ☆ | ☆ | ☆☆ | ☆ | ☆ |  | good |
| Park et al 2020  33124574 |  | ☆ | ☆ | ☆ | ☆☆ | ☆ |  |  | poor |
| Kwon et al 2020  33107238 |  | ☆ | ☆ | ☆ | ☆☆ | ☆ | ☆ |  | good |
| Ichinose et al 2020  32698892 |  | ☆ | ☆ | ☆ | ☆ | ☆ | ☆ |  | good |
| Ahn et al 2020  32635898 |  | ☆ | ☆ | ☆ | ☆☆ | ☆ | ☆ |  | good |
| Moroni et al 2020  32503858 | ☆ | ☆ | ☆ | ☆ | ☆☆ | ☆ | ☆ |  | good |
| Saleh et al 2020  32493152 |  | ☆ | ☆ | ☆ |  | ☆ | ☆ |  | poor |
| Tao et al 2020  32320985 |  | ☆ | ☆ | ☆ | ☆☆ | ☆ | ☆ |  | good |
| Tselios et al 2020  32238519 | ☆ | ☆ | ☆ | ☆ | ☆☆ | ☆ | ☆ | ☆ | good |
| Wang et al 2020  32228220 |  | ☆ | ☆ | ☆ | ☆ | ☆ |  |  | poor |
| Vajgel et al 2020  31801041 |  | ☆ | ☆ | ☆ | ☆☆ | ☆ | ☆ |  | good |
| Nakano et al 2019  31296139 | ☆ | ☆ | ☆ | ☆ | ☆☆ | ☆ | ☆ |  | good |
| Hu et al 2019  30975435 | ☆ | ☆ | ☆ | ☆ |  | ☆ | ☆ | ☆ | poor |
| Ichinose et al 2019  30700214 |  | ☆ | ☆ | ☆ | ☆☆ | ☆ | ☆ |  | good |
| Mackay et al 2019  30225865 | ☆ | ☆ | ☆ | ☆ | ☆☆ | ☆ | ☆ | ☆ | good |
| Yap et al  29509932 |  | ☆ | ☆ | ☆ |  | ☆ | ☆ |  | poor |
| Abdul Hamid et al 2023  L2026533724 |  | ☆ | ☆ | ☆ | ☆☆ | ☆ |  |  | poor |
| Gatto et al 2024  L2030257147 |  | ☆ | ☆ | ☆ | ☆ | ☆ | ☆ | ☆ | good |
| Yang et al 2022  L637226180 |  | ☆ | ☆ | ☆ | ☆☆ | ☆ | ☆ |  | good |
| Oh et al 2020  L2004007453 | ☆ | ☆ | ☆ | ☆ | ☆☆ | ☆ | ☆ |  | good |
| Das et al 2021  33611965 |  |  | ☆ | ☆ | ☆ | ☆ | ☆ |  | fair |
| Gopal et al 2023  37987842 |  | ☆ | ☆ | ☆ | ☆☆ | ☆ | ☆ |  | good |

**Supplementary Table 19** RoB2 for Randomized Control Trials

| Author | Risk of bias arising from the randomization process | Risk of bias due to deviations from the intended interventions | Missing outcome data | Risk of bias in measurement of the outcome | Risk of bias in selection of the reported result | **Overall risk of bias** |
| --- | --- | --- | --- | --- | --- | --- |
| Jourde-Chiche et al 2022  35725295 | Some concerns | Low | Low | Low | Low | Some |
| Rovin et al 2019  30420324 | Low | Low | Some concerns | Low | Low | Some concerns |
| Rovin et al 2021  33971155 | Low | Low | Low | Low | Low | Low |
| Zheng et al 2022  35353167 | Low | Low | Low | Low | Low | Low |
| Furie et al 2020  32937045 | Low | Low | Low | Low | Low | Low |
| Yu et al 2023  36058429 | Low | Low | Low | Low | Some | Some concerns |
| Atisha-Fregogo et al 2021  32755035 | Low | Low | High | Some concerns | Low | Some concerns |
| Fu et al 2022  35788493 | Some concerns | Low | Some concerns | Low | Low | Some concerns |
| Wang et al 2022  36288823 | High | Low | Low | Some concerns | Low | High |
| Bandhan et al 2022  34894070 | High | Some concerns | Low | Low | Low | High |
| Jayne et al 2022  35144924 | Low | Some concerns | Some concerns | Low | Some concerns | Some concerns |
| Chakravarty et al 2024  38301682 | Low | Low | Low | Some concerns | High | Some concerns |
| Bharati et al 2019  31571750 | High | High | Low | Some concerns | Some concerns | High |
| An et al 2019  30488367 | High | High | Low | Low | Some | High |
| Mok et al 2020  32448782 | Low | Low | Low | Low | Low | Low |
| Pal et al 2023 37881738 | Low | Some concerns | Low | Low | Low | Some concerns |
| Zhang et al 2020 33206751 | High | High | Low | Some concerns | Low | High |
| Zhang et al 2019 30426311 | Low | Low | Low | Low | Low | Low |
|  |  |  |  |  |  |  |
|  |  |  |  |  |  |  |

# References

1. Wang S, Spielman A, Ginsberg M, *et al.* Short- and Long-Term Progression of Kidney Involvement in Systemic Lupus Erythematosus Patients with Low-Grade Proteinuria. *Clin J Am Soc Nephrol*. 2022;17:1150–8. doi: 10.2215/CJN.01280122
2. Al Arfaj AS, Khalil N. Levels of Proteinuria and Renal Pathology in Systemic Lupus Erythematosus Patients. *Saudi J Kidney Dis Transpl*. 2023;34:154–60. doi: 10.4103/1319-2442.391894
3. Katsuyama E, Miyawaki Y, Sada K-E, *et al.* Association of explanatory histological findings and urinary protein and serum creatinine levels at renal biopsy in lupus nephritis: a cross-sectional study. *BMC Nephrol*. 2020;21:208. doi: 10.1186/s12882-020-01868-9
4. Carlucci PM, Li J, Fava A, *et al.* High incidence of proliferative and membranous nephritis in SLE patients with low proteinuria in the Accelerating Medicines Partnership. *Rheumatology (Oxford)*. 2022;61:4335–43. doi: 10.1093/rheumatology/keac067
5. De Rosa M, Rocha AS, De Rosa G, *et al.* Low-Grade Proteinuria Does Not Exclude Significant Kidney Injury in Lupus Nephritis. *Kidney Int Rep*. 2020;5:1066–8. doi: 10.1016/j.ekir.2020.04.005
6. Chedid A, Rossi GM, Peyronel F, et al. Low-Level Proteinuria in Systemic Lupus Erythematosus. Kidney Int Rep. 2020;5:2333–40. doi: 10.1016/j.ekir.2020.09.007
7. Gamaleldin SM, Alghazaly GM, Saad MA, et al. Urinary Sediments as Predictors of the Histopathology of Lupus Nephritis. Saudi J Kidney Dis Transpl. 2022;33:617–26. doi: 10.4103/1319-2442.389422
8. Gouda W, Abd Elaziz Alsaid A, Abbas AS, *et al.* Silent Lupus Nephritis: Renal Histopathological Profile and Early Detection with Urinary Monocyte Chemotactic Protein 1. *Open Access Rheumatol*. 2022;14:161–70. doi: 10.2147/OARRR.S373589
9. Rovin BH, Solomons N, Pendergraft WF, *et al.* A randomized, controlled double-blind study comparing the efficacy and safety of dose-ranging voclosporin with placebo in achieving remission in patients with active lupus nephritis. *Kidney Int*. 2019;95:219–31. doi: 10.1016/j.kint.2018.08.025
10. Rovin BH, Teng YKO, Ginzler EM, *et al.* Efficacy and safety of voclosporin versus placebo for lupus nephritis (AURORA 1): a double-blind, randomised, multicentre, placebo-controlled, phase 3 trial. *Lancet*. 2021;397:2070–80. doi: 10.1016/S0140-6736(21)00578-X
11. Saxena A, Ginzler EM, Gibson K, *et al.* Safety and Efficacy of Long-Term Voclosporin Treatment for Lupus Nephritis in the Phase 3 AURORA 2 Clinical Trial. *Arthritis Rheumatol*. 2024;76:59–67. doi: 10.1002/art.42657
12. Arriens C, Teng YKO, Ginzler EM, *et al.* Update on the Efficacy and Safety Profile of Voclosporin: An Integrated Analysis of Clinical Trials in Lupus Nephritis. *Arthritis Care Res (Hoboken)*. 2023;75:1399–408. doi: 10.1002/acr.25007
13. Furie R, Rovin BH, Houssiau F, *et al.* Two-Year, Randomized, Controlled Trial of Belimumab in Lupus Nephritis. *N Engl J Med*. 2020;383:1117–28. doi: 10.1056/NEJMoa2001180
14. Furie R, Rovin BH, Houssiau F, *et al.* Safety and Efficacy of Belimumab in Patients with Lupus Nephritis: Open-Label Extension of BLISS-LN Study. *Clin J Am Soc Nephrol*. 2022;17:1620–30. doi: 10.2215/CJN.02520322
15. Furie RA, Aroca G, Cascino MD, *et al.* B-cell depletion with obinutuzumab for the treatment of proliferative lupus nephritis: a randomised, double-blind, placebo-controlled trial. *Ann Rheum Dis*. 2022;81:100–7. doi: 10.1136/annrheumdis-2021-220920
16. Furie RA, Rovin BH, Garg JP, *et al.* Efficacy and Safety of Obinutuzumab in Active Lupus Nephritis. *N Engl J Med*. Published Online First: 7 February 2025. doi: 10.1056/NEJMoa2410965
17. Jayne D, Rovin B, Mysler EF, *et al.* Phase II randomised trial of type I interferon inhibitor anifrolumab in patients with active lupus nephritis. *Ann Rheum Dis*. 2022;81:496–506. doi: 10.1136/annrheumdis-2021-221478
18. Jayne D, Rovin B, Mysler E, *et al.* Anifrolumab in lupus nephritis: results from second-year extension of a randomised phase II trial. *Lupus Sci Med*. 2023;10:e000910. doi: 10.1136/lupus-2023-000910
19. Atisha-Fregoso Y, Malkiel S, Harris KM, *et al.* Phase II Randomized Trial of Rituximab Plus Cyclophosphamide Followed by Belimumab for the Treatment of Lupus Nephritis. *Arthritis Rheumatol*. 2021;73:121–31. doi: 10.1002/art.41466
20. Zheng Z, Zhang H, Peng X, *et al.* Effect of Tacrolimus vs Intravenous Cyclophosphamide on Complete or Partial Response in Patients With Lupus Nephritis: A Randomized Clinical Trial. *JAMA Netw Open*. 2022;5:e224492. doi: 10.1001/jamanetworkopen.2022.4492
21. Pal A, Chaudhury AR, Bhunia A, *et al.* A Randomized Controlled Trial Comparing Remission Induction with Modified Multitarget Therapy with Intravenous Cyclophosphamide in Proliferative Lupus Nephritis. *Indian J Nephrol*. 2023;33:340–7. doi: 10.4103/ijn.ijn_355_21
22. Zhang X, Liu P, Zhang Z. Analysis of the Clinical Effects of the Combination of Mycophenolate Mofetil with Either Tacrolimus or Cyclophosphamide. *Clinics (Sao Paulo)*. 2020;75:e1820. doi: 10.6061/clinics/2020/e1820
23. Zhang M, Qi C, Zha Y, *et al.* Leflunomide versus cyclophosphamide in the induction treatment of proliferative lupus nephritis in Chinese patients: a randomized trial. *Clin Rheumatol*. 2019;38:859–67. doi: 10.1007/s10067-018-4348-z
24. An Y, Zhou Y, Bi L, *et al.* Combined immunosuppressive treatment (CIST) in lupus nephritis: a multicenter, randomized controlled study. *Clin Rheumatol*. 2019;38:1047–54. doi: 10.1007/s10067-018-4368-8
25. Fu Q, Wu C, Dai M, *et al.* Leflunomide versus azathioprine for maintenance therapy of lupus nephritis: a prospective, multicentre, randomised trial and long-term follow-up. *Ann Rheum Dis*. 2022;81:1549–55. doi: 10.1136/ard-2022-222486
26. Mok CC, Ho LY, Ying SKY, *et al.* Long-term outcome of a randomised controlled trial comparing tacrolimus with mycophenolate mofetil as induction therapy for active lupus nephritis. *Ann Rheum Dis*. 2020;79:1070–6. doi: 10.1136/annrheumdis-2020-217178
27. Bandhan IH, Islam MN, Ahmad HI, *et al.* Outcome of low-dose prednisolone use for the induction of remission in lupus nephritis patients. *Int J Rheum Dis*. 2022;25:121–30. doi: 10.1111/1756-185X.14265
28. Bharati J, Rathi M, Ramachandran R, *et al.* Comparison of Two Steroid Regimens in Induction Therapy of Proliferative Lupus Nephritis: A Randomized Controlled Trial. *Indian J Nephrol*. 2019;29:373–5. doi: 10.4103/ijn.IJN_299_18
29. Gheet FS, Dawoud HE-S, El-Shahaby WA, *et al.* Hydroxychloroquine in children with proliferative lupus nephritis: a randomized clinical trial. *Eur J Pediatr*. 2023;182:1685–95. doi: 10.1007/s00431-023-04837-0
30. Arends EJ, Meziyerh S, Moes DJAR, *et al.* Voclosporin and the Antiviral Effect Against SARS-CoV-2 in Immunocompromised Kidney Patients. *Kidney Int Rep*. 2023;8:2654–64. doi: 10.1016/j.ekir.2023.09.003
31. Yu X, Chen N, Xue J, *et al.* Efficacy and Safety of Belimumab in Patients With Lupus Nephritis: Subgroup Analyses of a Phase 3 Randomized Trial in the East Asian Population. *Am J Kidney Dis*. 2023;81:294-306.e1. doi: 10.1053/j.ajkd.2022.06.013
32. Wang H, Li T, Sun F, *et al.* Safety and efficacy of the SGLT2 inhibitor dapagliflozin in patients with systemic lupus erythematosus: a phase I/II trial. *RMD Open*. 2022;8:e002686. doi: 10.1136/rmdopen-2022-002686
33. Rovin BH, Furie R, Teng YKO, *et al.* A secondary analysis of the Belimumab International Study in Lupus Nephritis trial examined effects of belimumab on kidney outcomes and preservation of kidney function in patients with lupus nephritis. *Kidney Int*. 2022;101:403–13. doi: 10.1016/j.kint.2021.08.027
34. Menn-Josephy H, Hodge LS, Birardi V, *et al.* Efficacy of Voclosporin in Proliferative Lupus Nephritis with High Levels of Proteinuria. *Clin J Am Soc Nephrol*. 2024;19:309–18. doi: 10.2215/CJN.0000000000000297
35. Anders H-J, Furie R, Malvar A, et al. Effect of belimumab on kidney-related outcomes in patients with lupus nephritis: post hoc subgroup analyses of the phase 3 BLISS-LN trial. Nephrol Dial Transplant. 2023;38:2733–42. doi: 10.1093/ndt/gfad167
36. Khosroshahi A, Tong D, Bao G, *et al.* Performance of Modified ALMS and BLISS Criteria with Standard of Care Treatment in Two US Health Care Systems. *Arthritis Care Res (Hoboken)*. 2023;75:1423–33. doi: 10.1002/acr.25025
37. Cooper Blenkinsopp S, Fu Q, Green Y, *et al.* Renal response at 2 years post biopsy to predict long-term renal survival in lupus nephritis: a retrospective analysis of the Hopkins Lupus Cohort. *Lupus Sci Med*. 2022;9:e000598. doi: 10.1136/lupus-2021-000598
38. Park D-J, Choi S-E, Xu H, *et al.* Uric acid as a risk factor for progression to chronic kidney disease in patients with lupus nephritis: results from the KORNET registry. *Clin Exp Rheumatol*. 2021;39:947–54. doi: 10.55563/clinexprheumatol/k4rdi7
39. Ichinose K, Kitamura M, Sato S, *et al.* Factors predictive of long-term mortality in lupus nephritis: a multicenter retrospective study of a Japanese cohort. *Lupus*. 2019;28:295–303. doi: 10.1177/0961203319826690
40. Hailu G-MT, Hussen SU, Getachew S, *et al.* Management practice and treatment outcomes of adult patients with Lupus Nephritis at the Renal Clinic of St. Paul’s Hospital Millennium Medical College, Addis Ababa, Ethiopia. *BMC Nephrol*. 2022;23:214. doi: 10.1186/s12882-022-02846-z
41. Enfrein A, Pirson V, Le Guern V, *et al.* Worse long-term renal outcome of lupus nephritis patients of African descent living in Europe. *RMD Open*. 2022;8:e002386. doi: 10.1136/rmdopen-2022-002386
42. Gatto M, Frontini G, Calatroni M, *et al.* Effect of Sustained Clinical Remission on the Risk of Lupus Flares and Impaired Kidney Function in Patients With Lupus Nephritis. *Kidney Int Rep*. 2024;9:1047–56. doi: 10.1016/j.ekir.2024.01.016
43. Zhang D, Sun F, Chen J, *et al.* Four trajectories of 24-hour urine protein levels in real-world lupus nephritis cohorts. *RMD Open*. 2023;9:e002930. doi: 10.1136/rmdopen-2022-002930
44. Perez-Arias AA, Márquez-Macedo SE, Pena-Vizcarra OR, *et al.* The influence of repeated flares in response to therapy and prognosis in lupus nephritis. *Nephrol Dial Transplant*. 2023;38:884–93. doi: 10.1093/ndt/gfac304
45. Zavala-Miranda MF, Perez-Arias AA, Márquez-Macedo SE, *et al.* Characteristics and outcomes of a Hispanic lupus nephritis cohort from Mexico. *Rheumatology (Oxford)*. 2023;62:1136–44. doi: 10.1093/rheumatology/keac407
46. Luo H, Zhou Y, Chen G, *et al.* Response to therapy at 6 months predicts long-term renal outcome in lupus nephritis with poor kidney function. *Lupus Sci Med*. 2022;9:e000773. doi: 10.1136/lupus-2022-000773
47. Jeon H, Lee J, Ju JH, *et al.* Chronic kidney disease in Korean patients with lupus nephritis: over a 35-year period at a single center. *Clin Rheumatol*. 2022;41:1665–74. doi: 10.1007/s10067-021-06030-w
48. Vajgel G, Oliveira CBL, Costa DMN, *et al.* Initial renal histology and early response predict outcomes of Brazilian lupus nephritis patients. *Lupus*. 2020;29:83–91. doi: 10.1177/0961203319890681
49. Moroni G, Gatto M, Tamborini F, *et al.* Lack of EULAR/ERA-EDTA response at 1 year predicts poor long-term renal outcome in patients with lupus nephritis. *Ann Rheum Dis*. 2020;79:1077–83. doi: 10.1136/annrheumdis-2020-216965
50. Zen M, Fuzzi E, Loredo Martinez M, *et al.* Immunosuppressive therapy withdrawal after remission achievement in patients with lupus nephritis. *Rheumatology (Oxford)*. 2022;61:688–95. doi: 10.1093/rheumatology/keab373
51. Pirson V, Enfrein A, Houssiau FA, *et al.* Absence of renal remission portends poor long-term kidney outcome in lupus nephritis. *Lupus Sci Med*. 2021;8:e000533. doi: 10.1136/lupus-2021-000533
52. Kapsia E, Marinaki S, Michelakis I, *et al.* Predictors of Early Response, Flares, and Long-Term Adverse Renal Outcomes in Proliferative Lupus Nephritis: A 100-Month Median Follow-Up of an Inception Cohort. *J Clin Med*. 2022;11:5017. doi: 10.3390/jcm11175017
53. Mackay M, Dall’Era M, Fishbein J, *et al.* Establishing Surrogate Kidney End Points for Lupus Nephritis Clinical Trials: Development and Validation of a Novel Approach to Predict Future Kidney Outcomes. *Arthritis Rheumatol*. 2019;71:411–9. doi: 10.1002/art.40724
54. Braga FNHF, das Chagas Medeiros MM, Junior ABV, *et al.* Proteinuria and serum creatinine after 12 months of treatment for lupus nephritis as predictors of long-term renal outcome: a case-control study. *Adv Rheumatol*. 2022;62:2. doi: 10.1186/s42358-021-00232-1
55. Farinha F, Barreira S, Couto M, *et al.* Risk of chronic kidney disease in 260 patients with lupus nephritis-analysis of a nationwide multicentre cohort with up to 35 years of follow-up. *Rheumatology (Oxford)*. 2024;keae236. doi: 10.1093/rheumatology/keae236
56. Tian N, Zhou Q, Yin P, *et al.* Long-Term Kidney Prognosis and Pathological Characteristics of Late-Onset Lupus Nephritis. *Front Med (Lausanne)*. 2022;9:882692. doi: 10.3389/fmed.2022.882692
57. Mejia-Vilet JM, Shapiro JP, Zhang XL, *et al.* Association Between Urinary Epidermal Growth Factor and Renal Prognosis in Lupus Nephritis. *Arthritis Rheumatol*. 2021;73:244–54. doi: 10.1002/art.41507
58. Kharouf F, Li Q, Whittall Garcia LP, *et al.* Short and Long-Term Outcomes of Patients with Pure Membranous Lupus Nephritis Compared to Patients with Proliferative Disease. *Rheumatology (Oxford)*. 2024;keae436. doi: 10.1093/rheumatology/keae436
59. Abdul Hamid SK, Elshazly A, Faisal YAE, *et al.* Renal arterial resistive index as a prognostic marker in lupus nephritis patients. *Nefrologia (Engl Ed)*. 2024;44:373–81. doi: 10.1016/j.nefroe.2024.04.006
60. Jeon H, Lee J, Moon S-J, *et al.* Predictors of renal relapse in Koreans with lupus nephritis after achieving complete response: a 35-years of experience at a single center. *Korean J Intern Med*. 2024;39:347–59. doi: 10.3904/kjim.2023.255
61. Zoshima T, Hara S, Suzuki K, *et al.* Long-term outcomes of lupus nephritis with low-level proteinuria: a multicentre, retrospective study. *Rheumatology (Oxford)*. 2024;63:3074–9. doi: 10.1093/rheumatology/kead624
62. Rodelo J, Aguirre L, Ortegón K, *et al.* Predicting kidney outcomes among Latin American patients with lupus nephritis: The prognostic value of interstitial fibrosis and tubular atrophy and tubulointerstitial inflammation. *Lupus*. 2023;32:411–23. doi: 10.1177/09612033231151597
63. Frontini G, Tamborini F, Porata G, *et al.* Rate and predictors of chronic organ damage accrual in active lupus nephritis: a single centre experience over 18 years of observation. *Clin Exp Rheumatol*. 2022;40:872–81. doi: 10.55563/clinexprheumatol/ig0lu0
64. Zhang T, Wang M, Zhang J, *et al.* Association between tubulointerstitial CD8+T cells and renal prognosis in lupus nephritis. *Int Immunopharmacol*. 2021;99:107877. doi: 10.1016/j.intimp.2021.107877
65. Ahn SS, Yoo J, Jung SM, *et al.* Comparison of clinical features and outcomes between patients with early and delayed lupus nephritis. *BMC Nephrol*. 2020;21:258. doi: 10.1186/s12882-020-01915-5
66. Kang Y, Zuo Y, He M, *et al.* Clinical predictive model to estimate probability of remission in patients with lupus nephritis. *Int Immunopharmacol*. 2022;110:108966. doi: 10.1016/j.intimp.2022.108966
67. Katsuyama E, Miyawaki Y, Sada K-E, *et al.* Association of explanatory histological findings and urinary protein and serum creatinine levels at renal biopsy in lupus nephritis: a cross-sectional study. *BMC Nephrol*. 2020;21:208. doi: 10.1186/s12882-020-01868-9
68. Gomez Mendez LM, Cascino MD, Katsumoto TR, *et al.* Outcome of participants with nephrotic syndrome in combined clinical trials of lupus nephritis. *Lupus Sci Med*. 2019;6:e000308. doi: 10.1136/lupus-2018-000308
69. Suttichet TB, Kittanamongkolchai W, Phromjeen C, *et al.* Urine TWEAK level as a biomarker for early response to treatment in active lupus nephritis: a prospective multicentre study. *Lupus Sci Med*. 2019;6:e000298. doi: 10.1136/lupus-2018-000298
70. Zhao L, Wang W, Wu L, *et al.* Combination of anti-SSA/Ro60 and anti-dsDNA serotype is predictive of belimumab renal response in patients with lupus nephritis. *Lupus Sci Med*. 2024;11:e001156. doi: 10.1136/lupus-2024-001156
71. Xia W, Deng J, Zhuang L, *et al.* Risk factors for acute kidney injury and kidney relapse in patients with lupus podocytopathy. *Clin Kidney J*. 2024;17:sfae148. doi: 10.1093/ckj/sfae148
72. Izmirly PM, Kim MY, Carlucci PM, *et al.* Longitudinal patterns and predictors of response to standard-of-care therapy in lupus nephritis: data from the Accelerating Medicines Partnership Lupus Network. *Arthritis Res Ther*. 2024;26:54. doi: 10.1186/s13075-024-03275-z
73. Chen Y, Wu X, Chen X, *et al.* Correlations of baseline neutrophil-lymphocyte ratio with prognosis of patients with lupus nephritis: A single-center experience. *Rheumatol Immunol Res*. 2023;4:196–203. doi: 10.2478/rir-2023-0029
74. Zoshima T, Hara S, Suzuki K, *et al.* Long-term outcomes of lupus nephritis with low-level proteinuria: a multicentre, retrospective study. *Rheumatology (Oxford)*. 2024;63:3074–9. doi: 10.1093/rheumatology/kead624
75. Márquez-Macedo SE, Perez-Arias AA, Pena-Vizcarra ÓR, *et al.* Predictors of treatment outcomes in lupus nephritis with severe acute kidney injury and requirement of dialytic support. *Clin Rheumatol*. 2023;42:2115–23. doi: 10.1007/s10067-023-06629-1
76. Gopal A, Kavadichanda C, Bairwa D, *et al.* Performance of Clinical and Biochemical Parameters in Identifying Renal Histopathology and Predictors of One-Year Renal Outcome in Lupus Nephritis-A Single Centre Study from India. *Diagnostics (Basel)*. 2022;12:3163. doi: 10.3390/diagnostics12123163
77. Zhang J, Song H, Li D, *et al.* Role of clinicopathological features for the early prediction of prognosis in lupus nephritis. *Immunol Res*. 2021;69:285–94. doi: 10.1007/s12026-021-09201-8
78. Luís MSF, Bultink IEM, da Silva JAP, *et al.* Early predictors of renal outcome in patients with proliferative lupus nephritis: a 36-month cohort study. *Rheumatology (Oxford)*. 2021;60:5134–41. doi: 10.1093/rheumatology/keab126
79. Nakano M, Kubo K, Shirota Y, *et al.* Delayed lupus nephritis in the course of systemic lupus erythematosus is associated with a poorer treatment response: a multicentre, retrospective cohort study in Japan. *Lupus*. 2019;28:1062–73. doi: 10.1177/0961203319860200
80. Fan Y, Kang D, Chen Z, *et al.* Clinicopathological characteristics and outcomes of lupus nephritis patients with scanty immune depositions in kidney biopsies. *J Nephrol*. 2023;36:2345–54. doi: 10.1007/s40620-023-01622-y
81. Whittall-Garcia LP, Naderinabi F, Gladman DD, *et al.* Circulating neutrophil extracellular trap remnants as a biomarker to predict outcomes in lupus nephritis. *Lupus Sci Med*. 2024;11:e001038. doi: 10.1136/lupus-2023-001038
82. Li S, Luo Q, Fan Y, *et al.* Clinicopathological Characteristics and Prognosis of Lupus Nephritis Patients with Acute Kidney Injury. *Am J Nephrol*. 2023;54:536–45. doi: 10.1159/000533847
83. Duran E, Yıldırım T, Taghiyeva A, *et al.* Differences and similarities of proliferative and non-proliferative forms of biopsy-proven lupus nephritis: Single centre, cross-disciplinary experience. *Lupus*. 2022;31:1147–56. doi: 10.1177/09612033221106305
84. Duan T, Zhu X, Zhao Q, *et al.* Association of Bowman’s capsule rupture with prognosis in patients with lupus nephritis. *J Nephrol*. 2022;35:1193–204. doi: 10.1007/s40620-022-01316-x
85. Moroni G, Porata G, Raffiotta F, *et al.* Beyond ISN/RPS Lupus Nephritis Classification: Adding Chronicity Index to Clinical Variables Predicts Kidney Survival. *Kidney360*. 2022;3:122–32. doi: 10.34067/KID.0005512021
86. Qiu S, Zhang H, Yu S, *et al.* Clinical manifestations, prognosis, and treat-to-target assessment of pediatric lupus nephritis. *Pediatr Nephrol*. 2022;37:367–76. doi: 10.1007/s00467-021-05164-y
87. Liu P, Peng Z, Xiang Y, *et al.* Causes and predictors of mortality from lupus nephritis in Southern Hunan, China. *Mod Rheumatol*. 2022;32:338–44. doi: 10.1080/14397595.2021.1920097
88. Strufaldi FL, Menezes Neves PDM de M, Dias CB, *et al.* Renal thrombotic microangiopathy associated to worse renal prognosis in Lupus Nephritis. *J Nephrol*. 2021;34:1147–56. doi: 10.1007/s40620-020-00938-3
89. Saleh M, Eltoraby EE, Tharwat S, *et al.* Clinical and histopathological features and short-term outcomes of lupus nephritis: a prospective study of 100 Egyptian patients. *Lupus*. 2020;29:993–1001. doi: 10.1177/0961203320928424
90. Tselios K, Gladman DD, Su J, *et al.* Advanced Chronic Kidney Disease in Lupus Nephritis: Is Dialysis Inevitable? *J Rheumatol*. 2020;47:1366–73. doi: 10.3899/jrheum.191064
91. Liao Y-W, Chen Y-M, Hsieh T-Y, *et al.* Renal Histopathology Associated With Kidney Failure and Mortality in Patients With Lupus Nephritis: A Long-Term Real-World Data Study. *J Rheumatol*. 2023;50:1127–35. doi: 10.3899/jrheum.220345
92. Kwon OC, Park JH, Lee SW, *et al.* Worse Renal Presentation and Prognosis in Initial-Onset Lupus Nephritis than Early-Onset Lupus Nephritis. *Yonsei Med J*. 2020;61:951–7. doi: 10.3349/ymj.2020.61.11.951
93. Ichinose K, Kitamura M, Sato S, *et al.* Comparison of complete renal response and mortality in early- and late-onset lupus nephritis: a multicenter retrospective study of a Japanese cohort. *Arthritis Res Ther*. 2020;22:175. doi: 10.1186/s13075-020-02271-3
94. Rong R, Wen Q, Wang Y, *et al.* Prognostic significance of hypertension at the onset of lupus nephritis in Chinese patients: prevalence and clinical outcomes. *J Hum Hypertens*. 2022;36:153–62. doi: 10.1038/s41371-021-00492-w
95. Hu J, Zhu M, Wang J, *et al.* The clinicopathological features and renal prognostic factors in pure membranous lupus nephritis-a large series cohort study from China. *Lupus*. 2024;33:192–200. doi: 10.1177/09612033231225345
96. Gomes MF, Mardones C, Xipell M, *et al.* The extent of tubulointerstitial inflammation is an independent predictor of renal survival in lupus nephritis. *J Nephrol*. 2021;34:1897–905. doi: 10.1007/s40620-021-01007-z
97. Reátegui-Sokolova C, Ugarte-Gil MF, Harvey GB, *et al.* Predictors of renal damage in systemic lupus erythematous patients: data from a multiethnic, multinational Latin American lupus cohort (GLADEL). *RMD Open*. 2020;6:e001299. doi: 10.1136/rmdopen-2020-001299
98. Liu R, Peng Y, Ye H, *et al.* Peripheral Eosinophil Count Associated with Disease Activity and Clinical Outcomes in Hospitalized Patients with Lupus Nephritis. *Nephron*. 2023;147:408–16. doi: 10.1159/000528486
99. Xue L, Shi Y, Zhang J, *et al.* Correlations of peripheral blood neutrophil-lymphocyte ratio and lymphocyte-monocyte ratio with renal function and prognosis in patients with lupus nephritis. *Am J Transl Res*. 2022;14:336–42.
100. Rossi GM, Maggiore U, Peyronel F, *et al.* Persistent Isolated C3 Hypocomplementemia as a Strong Predictor of End-Stage Kidney Disease in Lupus Nephritis. *Kidney Int Rep*. 2022;7:2647–56. doi: 10.1016/j.ekir.2022.09.012
101. Hu J, Zhu M, Wang J, *et al.* The clinicopathological features and renal prognostic factors in pure membranous lupus nephritis-a large series cohort study from China. *Lupus*. 2024;33:192–200. doi: 10.1177/09612033231225345
102. Fava A, Wagner CA, Guthridge CJ, *et al.* Association of Autoantibody Concentrations and Trajectories With Lupus Nephritis Histologic Features and Treatment Response. *Arthritis Rheumatol*. 2024;76:1611–22. doi: 10.1002/art.42941
103. Lin S, Zhang J, Chen B, *et al.* Role of crescents for lupus nephritis in clinical, pathological and prognosis: a single-center retrospective cohort study. *Eur J Med Res*. 2023;28:60. doi: 10.1186/s40001-023-01022-9
104. Yang X, Yuan Y, Shao X, *et al.* C4d as a Screening Tool and an Independent Predictor of Clinical Outcomes in Lupus Nephritis and IgA Nephropathy. *Front Med*. 2022;9:832998. doi: 10.3389/fmed.2022.832998
105. Mahmood SB, Aziz M, Malepati D, *et al.* Evaluating Sex Differences in the Characteristics and Outcomes of Lupus Nephritis: A Systematic Review and Meta-Analysis. *Glomerular Dis*. 2024;4:19–32. doi: 10.1159/000535981
106. Park E, Jung J, Min J, *et al.* Long-term outcomes and associated prognostic risk factors of childhood-onset lupus nephritis. *Kidney Res Clin Pract*. Published Online First: 18 December 2023. doi: 10.23876/j.krcp.23.032
107. Rodelo J, Aguirre L, Ortegón K, *et al.* Predicting kidney outcomes among Latin American patients with lupus nephritis: The prognostic value of interstitial fibrosis and tubular atrophy and tubulointerstitial inflammation. *Lupus*. 2023;32:411–23. doi: 10.1177/09612033231151597
108. Okamoto M, Kitamura M, Sato S, *et al.* Life prognosis and renal relapse after induction therapy in Japanese patients with proliferative and pure membranous lupus nephritis. *Rheumatology (Oxford)*. 2021;60:2333–41. doi: 10.1093/rheumatology/keaa599
109. Nunes MST, Barbosa Jorge L, Yu L, *et al.* Epidemiological, immunological, and treatment response profile of patients with lupus nephritis in Brazil. *Lupus*. 2024;33:650–8. doi: 10.1177/09612033241240869
110. Umeda R, Ogata S, Hara S, *et al.* Comparison of the 2018 and 2003 International Society of Nephrology/Renal Pathology Society classification in terms of renal prognosis in patients of lupus nephritis: a retrospective cohort study. *Arthritis Res Ther*. 2020;22:260. doi: 10.1186/s13075-020-02358-x
111. Wang S, Shang J, Xiao J, *et al.* Clinicopathologic characteristics and outcomes of lupus nephritis with positive antineutrophil cytoplasmic antibody. *Ren Fail*. 2020;42:244–54. doi: 10.1080/0886022X.2020.1735416
112. Tao J, Wang H, Yu X-J, *et al.* A Validation of the 2018 Revision of International Society of Nephrology/Renal Pathology Society Classification for Lupus Nephritis: A Cohort Study from China. *Am J Nephrol*. 2020;51:483–92. doi: 10.1159/000507213
113. Leatherwood C, Speyer CB, Feldman CH, *et al.* Clinical characteristics and renal prognosis associated with interstitial fibrosis and tubular atrophy (IFTA) and vascular injury in lupus nephritis biopsies. *Semin Arthritis Rheum*. 2019;49:396–404. doi: 10.1016/j.semarthrit.2019.06.002
114. Wang J, Lou W, Zhu M, *et al.* Prediction of treatment response in lupus nephritis using density of tubulointerstitial macrophage infiltration. *Front Immunol*. 2024;15:1321507. doi: 10.3389/fimmu.2024.1321507
115. Duong MD, Wang S, Schwartz D, *et al.* Total cortical interstitial inflammation predicts chronic kidney disease progression in patients with lupus nephritis. *Nephrol Dial Transplant*. 2023;38:1469–76. doi: 10.1093/ndt/gfac286
116. Gao Y, Wang B, Cao J, *et al.* Elevated Urinary Neutrophil Gelatinase-Associated Lipocalin Is a Biomarker for Lupus Nephritis: A Systematic Review and Meta-Analysis. *Biomed Res Int*. 2020;2020:2768326. doi: 10.1155/2020/2768326
117. Whittall-Garcia LP, Naderinabi F, Gladman DD, *et al.* Circulating neutrophil extracellular trap remnants as a biomarker to predict outcomes in lupus nephritis. *Lupus Sci Med*. 2024;11:e001038. doi: 10.1136/lupus-2023-001038
118. Ayoub I, Wolf BJ, Geng L, *et al.* Prediction models of treatment response in lupus nephritis. *Kidney Int*. 2022;101:379–89. doi: 10.1016/j.kint.2021.11.014
119. Wu Q, Qin Y, Shi M, *et al.* Diagnostic significance of circulating miR-485-5p in patients with lupus nephritis and its predictive value evaluation for the clinical outcomes. *J Chin Med Assoc*. 2021;84:491–7. doi: 10.1097/JCMA.0000000000000522
120. Malvar A, Alberton V, Lococo B, *et al.* Kidney biopsy-based management of maintenance immunosuppression is safe and may ameliorate flare rate in lupus nephritis. *Kidney Int*. 2020;97:156–62. doi: 10.1016/j.kint.2019.07.018
121. Das U, Patel R, Guditi S, *et al.* Correlation between the clinical remission and histological remission in repeat biopsy findings of quiescent proliferative lupus nephritis. *Lupus*. 2021;30:876–83. doi: 10.1177/0961203321995251
122. Lledó-Ibáñez GM, Xipell M, Ferreira M, *et al.* Kidney biopsy in lupus nephritis after achieving clinical renal remission: paving the way for renal outcome assessment. *Clin Kidney J*. 2022;15:2081–8. doi: 10.1093/ckj/sfac150
123. Gatto M, Radice F, Saccon F, *et al.* Clinical and histological findings at second but not at first kidney biopsy predict end-stage kidney disease in a large multicentric cohort of patients with active lupus nephritis. *Lupus Sci Med*. 2022;9:e000689. doi: 10.1136/lupus-2022-000689
124. Moroni G, Porata G, Raffiotta F, *et al.* Predictors of increase in chronicity index and of kidney function impairment at repeat biopsy in lupus nephritis. *Lupus Sci Med*. 2022;9:e000721. doi: 10.1136/lupus-2022-000721
125. Gupta KL, Bharati J, Anakutti H, *et al.* Contribution of Clinically Indicated Repeat Renal Biopsy in Indian Patients with Lupus Nephritis. *Indian J Nephrol*. 2020;30:377–81. doi: 10.4103/ijn.IJN_166_19
126. Parodis I, Adamichou C, Aydin S, *et al.* Per-protocol repeat kidney biopsy portends relapse and long-term outcome in incident cases of proliferative lupus nephritis. *Rheumatology (Oxford)*. 2020;59:3424–34. doi: 10.1093/rheumatology/keaa129

**
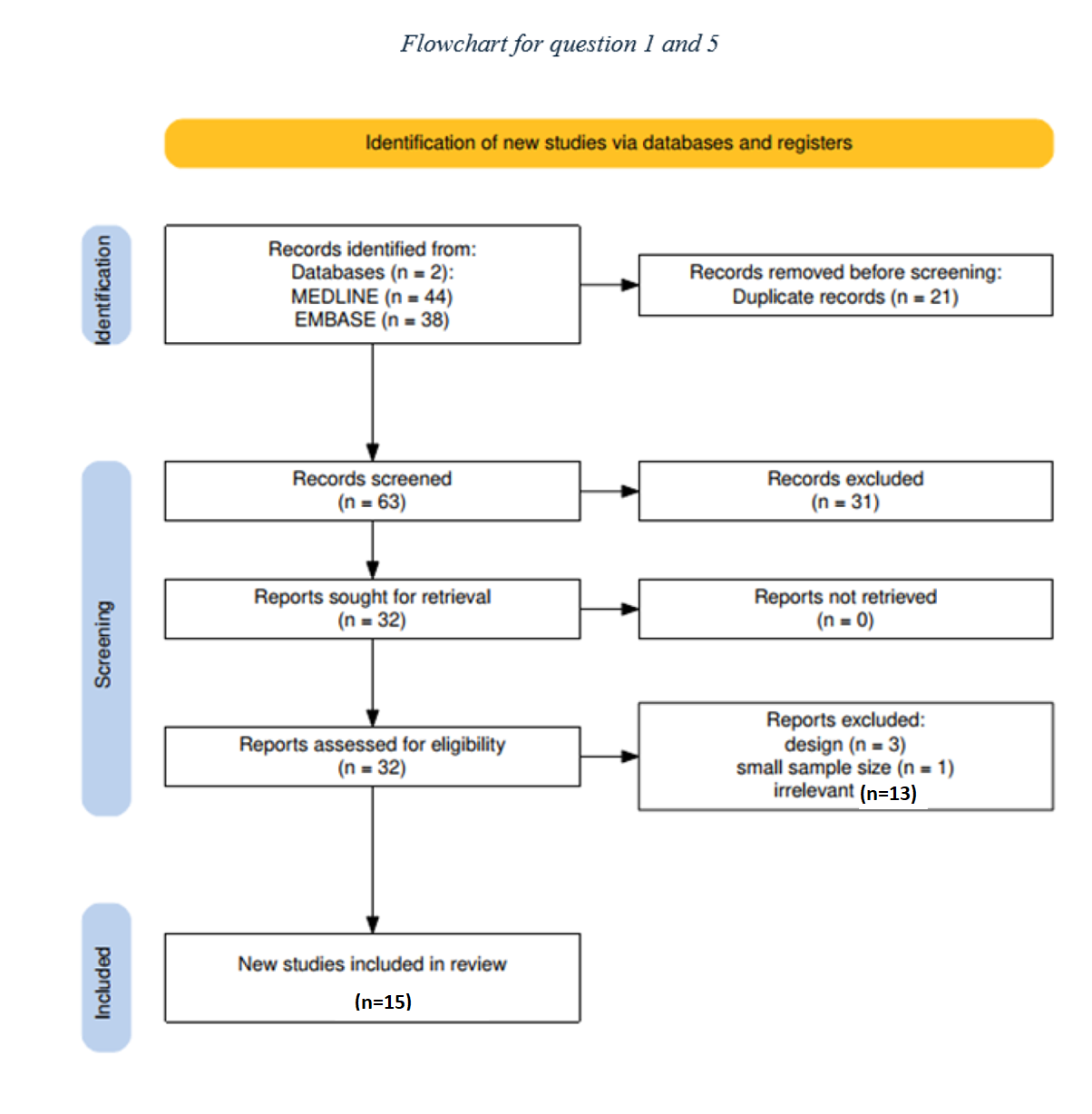
Figure 1 Flowchart for sections 1 and 6**

**Figure 2 Flowchart for sections 2,3 and 5**


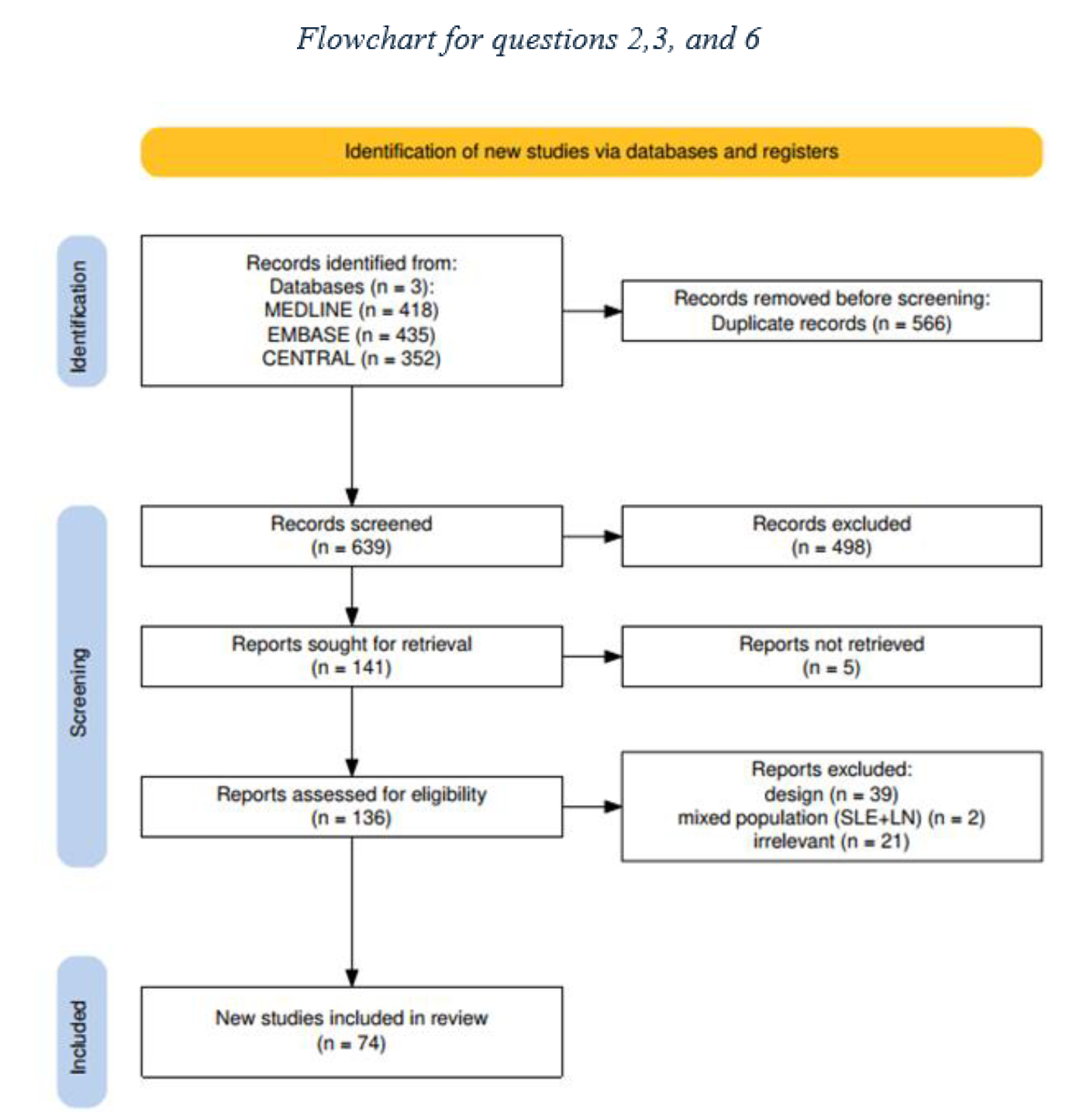


**Figure 3 Flowchart for section 4**
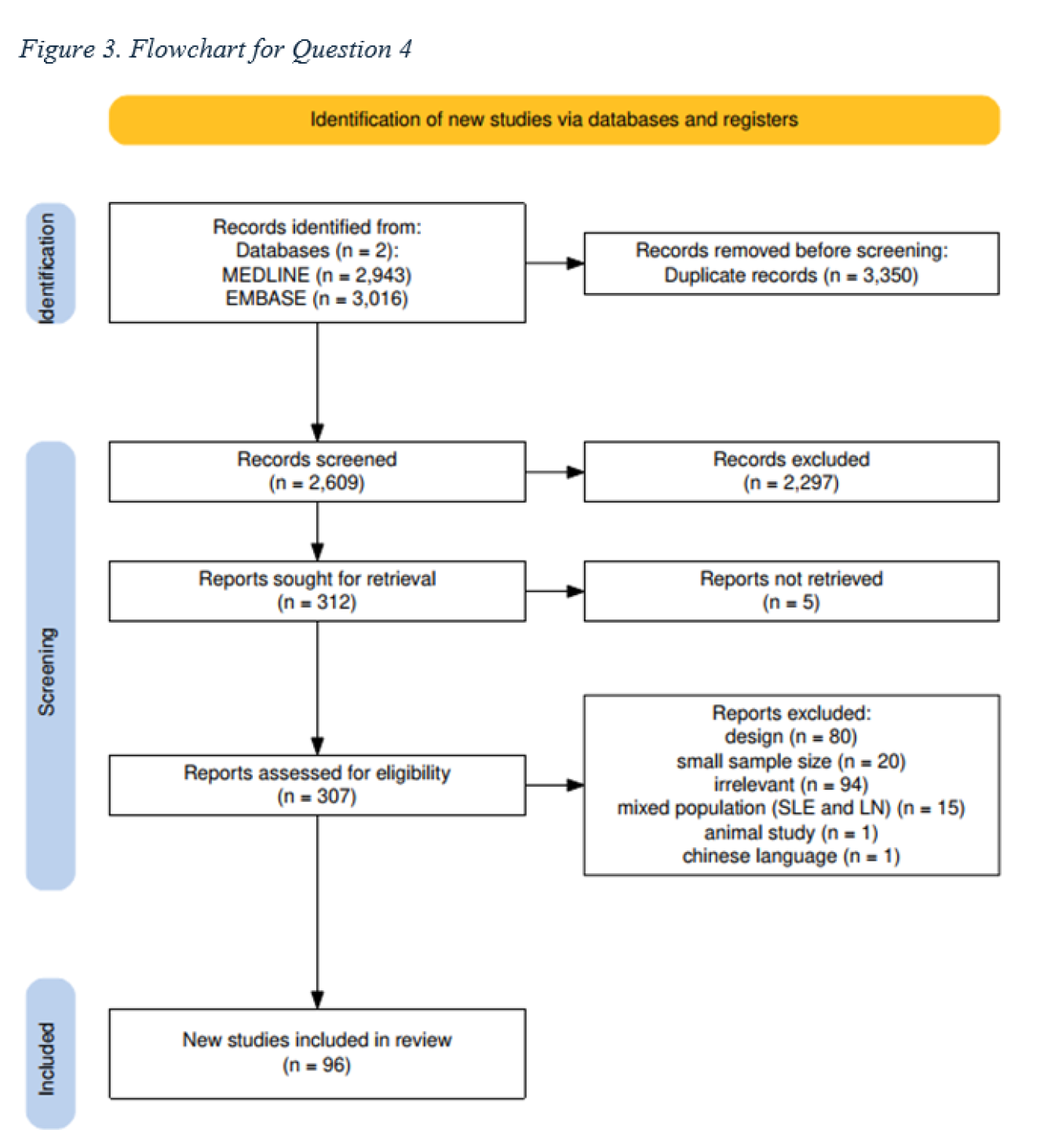

Supplement: Supplementary file 1 [file mmc1.docx]
